# Unveiling the Interconnected Dynamics of Mitochondrial Dysfunction Associated With Age-Related Cardiovascular Risk: A Cross-Sectional Pilot Study

**DOI:** 10.7759/cureus.82961

**Published:** 2025-04-25

**Authors:** Nikita Soni, Prasan Kaur, Vikas Gurjar, Arpit Bhargava, Rajnarayan Tiwari, Apoorva Chouksey, Rupesh K Srivastava, Pradyumna K Mishra

**Affiliations:** 1 Environmental Biotechnology, Genetics, and Molecular Biology, Indian Council of Medical Research (ICMR) - National Institute for Research in Environmental Health, Bhopal, IND; 2 Faculty of Science, Ram Krishna Dharmarth Foundation (RKDF) University, Bhopal, IND; 3 Epidemiology, Indian Council of Medical Research (ICMR) - National Institute for Research in Environmental Health, Bhopal, IND; 4 Biotechnology, All India Institute of Medical Sciences, New Delhi, New Delhi, IND

**Keywords:** cardiovascular aging, cardiovascular diseases, cellular senescence, circulating nucleic acids, epigenetic biomarkers, mitochondrial dysfunction, mitochondrial epigenetics, translational research

## Abstract

Aging, influenced by complex epigenetic mechanisms, significantly contributes to the progression of cardiovascular diseases (CVDs). This cross-sectional pilot study investigated mitochondrial-associated epigenetic stress responses in two age groups: Group I (18-38, n = 154), representing younger adults generally at lower risk for CVD, and Group II (39-65, n = 105), representing middle-aged and older adults with increased biological susceptibility. The age grouping was based on established physiological and cardiovascular risk transitions typically observed around age 40.

To assess age-related molecular variations, we examined key mitochondrial and metabolic parameters, including mitochondrial DNA (mtDNA) damage repair capacity, mtDNA copy number (mtDNAcn), methylation status, mitochondrial dynamics (fusion/fission), telomere length, expression of respiratory complex genes, levels of pro-inflammatory cytokines, and N-terminal pro-B-type natriuretic peptide (NT-proBNP) concentrations.

Our results indicated that the older group exhibited higher mtDNA methylation (r² = 0.5205, p < 0.0001), increased mtDNAcn, and elevated NT-proBNP levels, which also showed a weak positive correlation with mtDNA methylation (r² = 0.3218, p < 0.0001). Additionally, a strong negative correlation was observed between telomerase reverse transcriptase (TERT) expression and age (r² = 0.6070, p < 0.0001), suggesting a decline in telomeric maintenance with advancing age. Group II also showed altered inflammatory and telomeric profiles and a notable reduction in the expression of mitochondrial respiratory complex genes (ND6, COXI, ATPase 6 and 8), alongside increased expression of genes involved in mitochondrial stress response pathways.

We employed four machine learning models - Logistic Regression, Decision Tree, Random Forest, and Support Vector Machine (SVM) - for CVD risk prediction, using selected mitochondrial and metabolic features. All models demonstrated high classification accuracy, ranging from 0.920 to 1.0, with the Random Forest model achieving the highest accuracy of 0.984. These preliminary findings highlight distinct age-related molecular signatures and illustrate the potential of combining biomarkers with machine-learning approaches to improve cardiovascular risk prediction and therapeutic targeting in aging populations.

## Introduction

The global challenge of an aging population has attracted significant interest in recent years, resulting in an expanding array of research focused on improving both the safety and quality of life for elderly individuals. Epidemiological data indicate that 12% of the global population is over 60 years old, with projections rising to 22% by 2050 [1]. This demographic shift presents novel challenges to public health. Aging is an irreversible process marked by declining cellular and tissue functions, significantly increasing the risk of age-related diseases, particularly cardiovascular diseases (CVDs). CVDs are the leading cause of morbidity and mortality, with mortality rates of 35%-40% in individuals aged 40-60, 77%-80% in those aged 60-80, and over 80% for those above 80 [2]. Mitochondrial dysfunction is a central contributor to aging and cardiovascular pathophysiology [3].

Mitochondria not only generate cellular energy but also play essential roles in regulating oxidative stress, apoptosis, and inflammatory signaling. Age-related mitochondrial alterations include reduced mitochondrial DNA (mtDNA) integrity, impaired biogenesis, increased reactive oxygen species (ROS) production, and disrupted mitochondrial dynamics (fusion and fission) [4-6]. These changes compromise cellular homeostasis and drive inflammatory responses through pathways such as NF-κB signaling, which is known to enhance cytokine production (TNF-α (tumor necrosis factor-alpha) and IL-6 (interleukin-6)), and endothelial dysfunction [7,8]. Additionally, the upregulation of NF-κB has been consistently observed in age-associated inflammatory disorders [9,10].

The activation of the NF-κB-mediated inflammatory response is significantly driven by the formation of a Nod-like receptor pyrin domain-containing 3 (NLRP3) inflammasome, which contributes to atherosclerosis. In the aging process, persistent NLRP3 activity increases oxidative stress and mitochondrial dysfunction, leading to cellular damage and immunosenescence. This chronic inflammation, termed inflammaging, causes endothelial dysfunction and plays a critical role in the development of atherosclerosis and CVDs [11,12]. Furthermore, the disruption of mitochondria may trigger the activation of the integrated stress response (ISR), which is involved in the progression of cardiac dysfunction [13]. Aging induces pro-survival signaling, which results in mito-epigenetic modifications, altering mtDNA methylation and potentially increasing the risk of CVD due to impaired cardiomyocyte function [14]. Key enzymes such as TET2 (ten-eleven translocation methylcytosine dioxygenase 2) and DNMT (DNA methyltransferase) influence these changes, affecting gene methylation and microRNA (miRNA) profiles [15]. Dysregulation of mitochondrial-derived microRNAs (mitomiRs) impacts vascular smooth muscle cell proliferation, endothelial dysfunction, and inflammation, all of which are relevant to CVD pathogenesis [16].

Peripheral blood mononuclear cells (PBMCs) are ideal for assessing systemic changes and molecular signaling related to diseases because they are easily accessible [17]. Research on sepsis has shown that bioenergetic profiling of PBMCs correlates with mitochondrial function in other tissues and is associated with disease severity and prognosis. These cells reflect the body's responses to injury and illness, making them valuable for evaluating bioenergetics in translational research [18]. Characteristics of impaired mitophagy and mitochondrial quality control mechanisms, which are indicative of cardiac aging, can be seen in PBMCs through a loss of mitochondrial membrane potential and structural damage in patients with CVD. Therefore, elevated levels of circulating cell-free mtDNA may indicate mitochondrial damage/stress in cardiac cells [19,20]. Thus, assessing mitochondrial function in PBMCs offers a non-invasive approach to diagnosing CVD risk.

In this study, we investigated age-associated mitochondrial and epigenetic stress responses in PBMCs across two distinct age groups, aiming to identify molecular alterations that may indicate early susceptibility to CVD. We focused on mtDNA integrity, copy number, methylation status, mitochondrial dynamics, and circulating metabolic and inflammatory markers. Our central hypothesis was that aging induces measurable mitochondrial-epigenetic alterations in PBMCs that reflect systemic changes contributing to increased cardiovascular vulnerability.

## Materials and methods

Reagents and kits

All reagents utilized in this study were of the highest quality available. Lymphocytes were isolated employing HiSep™ (HiMedia Laboratories, Mumbai, India), along with 1× phosphate-buffered saline (PBS) (Cell Signaling Technology, Danvers, MA, USA). Whole-cell DNA was extracted using the PureLink Genomic DNA Mini Kit from Invitrogen, a subsidiary of Life Technologies (Thermo Fisher Scientific, Waltham, MA, USA). The isolation of total RNA was performed using the TRIzol® Reagent (Life Technologies - Thermo Fisher Scientific). For the measurement of oxidative stress, the CellROX® Deep Red Flow Cytometry assay kit (Thermo Fisher Scientific) was employed. The quantification of mitochondrial fission and fusion, signaling, and survival genes was conducted using the Luna® Universal One-Step RT-qPCR Kit (New England Biolabs, Ipswich, MA, USA) via real-time quantitative polymerase chain reaction (qPCR). The Luna® Universal qPCR Master Mix (New England Biolabs) was utilized to assess mtDNA methylation and DNMT1 levels, as well as the relative expression of mitomiRs and their target genes. For the evaluation of mitochondrial gene expression through conventional PCR, Taq 2X Master Mix (New England Biolabs) was employed. The examination of mtDNA methylation was performed utilizing BamHI restriction enzymes (Takara Bio Inc., Shiga, Japan), alongside the EpiJET Bisulfite Conversion Kit (Thermo Fisher Scientific). Furthermore, the profiling of mitomiRs involved the use of the Poly(A) Tailing Kit and the PureLink miRNA Isolation Kit (Invitrogen - Thermo Fisher Scientific). For gel electrophoresis, SeaKem® LE Agarose (Lonza, Basel, Switzerland) was employed, in conjunction with 50× TAE and 6X gel loading buffer (HiMedia Laboratories). Additionally, SYBR® Safe DNA gel stain (Invitrogen - Thermo Fisher Scientific), as well as 100 bp and 50 bp DNA ladders (HiMedia Laboratories and Promega Corporation, Madison, WI, USA), were utilized (Table 1).

**Table 1:** List of primers used for analysis of genes

S. No.	Primer	Primer sequence (5’->3’)	Size (bp)	Thermal condition
1	Drp1-F	GAATGACCAAGGTGCCTGTAG	191	95 °C for 1 min; (95 °C for 15 sec, 60.39 °C for 15 sec) × 35; (95 °C for 15 sec, 60 °C for 60 sec, 95 °C for 15 sec) × 1; 4 °C ∞
	Drp1-R	AGCTAGGGTTCTGCGACCAT		
2	Fis1-F	CTAGCTCCAGGGCCTGTTTGT	1979	95 °C for 1 min; (95 °C for 15 sec, 62.20 °C for 15 sec) × 35; (95 °C for 45 sec, 60 °C for 60 sec, 95 °C for 15 sec) × 1; 4 °C ∞
	Fis1-R	GGTGAAAGGACCCGTTTCCAG		
3	MFF-F	CAGGATCCATGAGTAAAGGAACAAGCAGTGA	755	95 °C for 1 min; (95 °C for 15 sec, 68.73 °C for 45 sec) × 35; (95 °C for 15 sec, 60 °C for 60 sec, 95 °C for 15 sec) × 1; 4 °C ∞
	MFF-R	TACTCGAGCTAGCGGCGAAACCAGAGC		
4	MFN1-F	GATGCACCGATGAAGTAAACGC	203	95 °C for 1 min; (95 °C for 15 sec, 60.84 °C for 15 sec) × 35; (95 °C for 15 sec, 60 °C for 60 sec, 95 °C for 15 sec) × 1; 4 °C ∞
5	MFN2-F	AGAGGCATCAGTGAGGTGCT	112	95 °C for 1 min; (95 °C for 15 sec, 60.59 °C for 15 sec) × 35; (95 °C for 45 sec, 60 °C for 60 sec, 95 °C for 15 sec) × 1; 4 °C ∞
	MFN2-R	GCAGAACTTTGTCCCAGAGC		
6	OPA1-F	GGCCAGCAAGATTAGCTACG	154	95 °C for 1 min; (95 °C for 15 sec, 60.59 °C for 15 sec) × 35; (95 °C for 45 sec, 60 °C for 60 sec, 95 °C for 15 sec) × 1; 4 °C ∞
	OPA1-R	ACAATGTCAGGCACAATCCA		
7	ND-6-F	CAAACAATGTTCAACCAGTAACCACTAC	281	95 °C for 2 min; (95 °C for 30 sec, 61.92 °C for 20 sec, 72 °C for 30 sec) × 35; 72 °C for 5 min; 4 °C ∞
	ND-6-R	ATATACTACAGCGATGGCTATTGAGGA		
8	Cyt-OX1-F	GACGTAGACACACGAGCATATTTCA	251	95 °C for 2 min; (95 °C for 30 sec, 62.3 °C for 20 sec, 72 °C for 30 sec) × 35; 72 °C for 5 min; 4 °C ∞
	Cyt-OX1-R	AGGACATAGTGGAAGTGAGCTACAAC		
9	ATPase-6-F	TAGCCATACACAACACTAAAGGACGA	201	95 °C for 2 min; (95 °C for 30 sec, 60.8 °C for 15 sec, 72 °C for 30 sec) × 35; 72 °C for 5 min; 4 °C ∞
	ATPase-6-R	GGGCATTTTTAATCTTAGAGCGAAA		
10	ATPase-8-F	ATGGCCCACCATAATTACCC	141	95 °C for 2 min; (95 °C for 30 sec, 57 °C for 15 sec, 72 °C for 30 sec) × 35; 72 °C for 5 min; 4 °C ∞
	ATPase-8-R	CATTTTGGTTCTCAGGGTTTG		
11	OMA1-F	GGGCCATTTGTCCTCGAGAT	137	95 °C for 1 min; (95 °C for 15 sec, 60.44 °C for 15 sec) × 35; (95 °C for 15 sec, 60 °C for 60 sec, 95 °C for 15 sec) × 1; 4 °C ∞
	OMA1-R	TGCAGCAAGCAGTAGTCCAA		
12	DELE1-F	TTCCAGCTCAGTGTTTCCATC	256	95 °C for 1 min; (95 °C for 15 sec, 58.28 °C for 15 sec) × 35; (95 °C for 15 sec, 60 °C for 60 sec, 95 °C for 15 sec) × 1; 4 °C ∞
	DELE1-R	GTAGTAGGCACCTGGCATAG		
13	HRI-F	GCAATTCCATATGCAGGGGGGCAACTCCGG	1767	95 °C for 1 min; (95 °C for 15 sec, 69.83 °C for 90 sec) × 35; (95 °C for 75 sec, 60 °C for 60 sec, 95 °C for 15 sec) × 1; 4 °C ∞
	HRI-R	CCTTGCTCGAGGAAAAGTTCACTCTGC		
14	DNMT 1-F	ACCGCTTCTACTTCCTCGAGGCCTA	335	95 °C for 2 min; (95 °C for 30 sec, 57 °C for 30 sec, 72 °C for 30 sec) × 35; 72 °C for 5 min; 4 °C ∞
	DNMT 1-R	GTTGCAGTCCTCTGTGAACACTGTGG		
15	DNMT 3A-F	CACACAGAAGCATATCCAGGAGTG	551	95 °C for 2 min; (95 °C for 30 sec, 53 °C for 30 sec, 72 °C for 30 sec) × 35; 72 °C for 5 min; 4 °C ∞
	DNMT3A-R	AGTGGACTGGGAAACCAAATACCC		
16	DNMT 3B-F	AATGTGAATCCAGCCAGGAAAGGC	191	95 °C for 2 min; (95 °C for 30 sec, 53 °C for 30 sec, 72 °C for 30 sec) × 35; 72 °C for 5 min; 4 °C ∞
	DNMT 3B-R	ACTGGATTACACTCCAGGAACCGT		
17	TFAM-F	AGCTCAGAACCCAGATGC	116	95 °C for 2 min; (95 °C for 30 sec, 57.33 °C for 30 sec, 72 °C for 30 sec) × 35; 72 °C for 5 min; 4 °C ∞
	TFAM-R	CCACTCCGCCCTATAAGC		
18	D-loop1-F	AAATCTATCACCCTATTAAC	292	95 °C for 1 min; (95 °C for 15 sec, 55 °C for 30 sec, 95 °C for 15 sec) × 35; (95 °C for 15 sec, 60 °C for 60 sec, 95 °C for 15 sec) × 1; 4 °C ∞
	D-loop1-R	GTGGAAATTTTTTGTTATGATGT		
19	D-loop2- F	CATAACAAAAAATTTCCACCAAAC	179	95 °C for 1 min; (95 °C for 15 sec, 55 °C for 30 sec, 95 °C for 15 sec) × 35; (95 °C for 15 sec, 60 °C for 60 sec, 95 °C for 15 sec) × 1; 4 °C ∞
	D-loop2- R	GGGAAAATAATGTGTTAGTT		
20	12s-TF-F	TTTATATAACTTACCTCCTC	188	95 °C for 1 min; (95 °C for 15 sec, 55 °C for 30 sec, 95 °C for 15 sec) × 35; (95 °C for 15 sec, 60 °C for 60 sec, 95 °C for 15 sec) × 1; 4 °C ∞
	12s-TF-R	GTGTTTGATGTTTGTTTTTTTTG		
21	16S-F	AATAAATTTATAGGTTTTTAAATTATTAAAT	152	95 °C for 1 min; (95 °C for 15 sec, 55 °C for 30 sec, 95 °C for 15 sec) × 35; (95 °C for 15 sec, 60 °C for 60 sec, 95 °C for 15 sec) × 1; 4 °C ∞
	16S-R	TAACTAATAAAATCTTAACATATACTACTC		
22	Cyt B-F	GGTATTATTTTTTTGTTTGTAATTATAGTA	110	95 °C for 1 min; (95 °C for 15 sec, 53 °C for 30 sec, 95 °C for 15 sec) × 35; (95 °C for 15 sec, 60 °C for 60 sec, 95 °C for 15 sec) × 1; 4 °C ∞
	Cyt B-R	CCTCAAATTCATTAAACTAAATCTATCC		
23	TET1-F	CAGAACCTAAACCACCCGTG	141	95 °C for 2 min; (95 °C for 30 sec, 60.1 °C for 15 sec, 72 °C for 30 sec) × 35; 72 °C for 5 min; 4 °C ∞
	TET1-R	TGCTTCGTAGCGCCATTGTAA		
24	TET2-F	GATAGAACCAACCATGTTGAGGG	95	95 °C for 2 min; (95 °C for 30 sec, 60.7 °C for 15 sec, 72 °C for 30 sec) × 35; 72 °C for 5 min; 4 °C ∞
	TET2-R	TGGAGCTTTGTAGCCAGAGGT		
25	TET3-F	TCCAGCAACTCCTAGAACTGAG	169	95 °C for 2 min; (95 °C for 30 sec, 60.4 °C for 15 sec, 72 °C for 30 sec) × 35; 72 °C for 5 min; 4 °C ∞
	TET3-R	AGGCCGCTTGAATACTGACTG		
26	IL-6-F	AATGTGAATCCAGCCAGGAAAGGC	188	95 °C for 2 min; (95 °C for 30 sec, 60.105 °C for 15 sec, 72 °C for 15 sec) × 35; 72 °C for 5 min; 4 °C ∞
	IL-6-R	ACTGGATTACACTCCAGGAACCGT		
27	TNF-α-F	CAGAACCTAAACCACCCGTG	157	95 °C for 2 min; (95 °C for 30 sec, 57.54 °C for 15 sec, 72 °C for 15 sec) × 35; 72 °C for 5 min; 4 °C ∞
	TNF-α-R	TGCTTCGTAGCGCCATTGTAA		
28	BamH1	G/GATCC		37 °C for 4 h
		CCTAG/G		

Study design

In the current study, we investigated age-related mitochondrial-mediated stress responses and their potential contribution to CVD susceptibility by stratifying participants into two distinct age groups: Group I (n = 154; individuals aged 18-38 years) and Group II (n = 105; individuals aged 39-65 years). The study followed a cross-sectional design and utilized PBMCs to assess mitochondrial and immunological responses due to their effectiveness in representing systemic physiological changes in vitro. A total of 259 blood samples were collected through random sampling from both urban and rural regions across various districts in Madhya Pradesh, India, ensuring a more representative population sample. All procedures were approved by the Institutional Human Ethics Committee (IHEC) of ICMR-National Institute for Research in Environmental Health (NIREH), Bhopal, and conducted under funding support from the Department of Health Research (DHR), Ministry of Health and Family Welfare (MoHFW), Government of India. Participants with a known history of CVD, diabetes, metabolic disorders, BP, or other chronic illnesses were excluded from the study to minimize confounding variables. For BP, in Groups I and II, the variables such as “Low Blood Pressure” and “High Blood Pressure” were categorized. Specifically, blood pressure was categorized based on established clinical thresholds: systolic BP <90 mmHg was classified as low, 90-139 mmHg as normal, and ≥140 mmHg as high. Approximately 10 mL of ethylenediaminetetraacetic acid (EDTA)-anticoagulated blood was collected from each participant, and informed consent was obtained as per Institutional Review Board guidelines. Samples were transported under cold chain conditions (2-8°C) and processed within two to three hours of collection. PBMCs were isolated and stored at -80°C until further analysis.

Analysis of mitochondrial oxidative DNA damage

To assess oxidative damage, the presence of oxidized purine bases (commonly deoxyguanosine) was examined by using the formamidopyrimidine glycosylase (FPG) digestion method with a negative control. The level of oxidative damage in mtDNA was observed as increased quantities of oxidized purine nucleotides. Furthermore, intracellular oxygen radical species and the generation of ROS were assessed by measuring the fluorescence intensity levels of 20,70-dichlorofluorescin (DCF) through 5-(and-6)-chloromethyl-20,70-dichlorodihydrofluorescein diacetate acetyl ester (CM-H2DCFDA) labeling and by following the protocol discussed elsewhere [21].

Aging-induced mitochondrial epigenetic alterations

To explore mitochondrial dysfunction, the whole DNA, RNA, and protein were extracted. The expression levels of mitochondrial fission and fusion genes (Drp1, Fis1, Mff, MFN1, MFN2, and OPA1), as well as the mitochondrial genes (MT-ATPase6, MT-ATPase8, MT-CO1, and MT-ND6), were assessed to understand mitochondrial dynamics and functioning. Furthermore, we analyzed the expression levels of genes associated with the ISR (DELE1, HRI, and OMA1) and the repair process (POLG, OGG, and APE). We also evaluated mtDNA methylation in the D-loop, 12S, cytochrome B, and 16S rRNA regions, as well as mtDNA methyltransferase 1 (DNMT1, DNMT3a, and DNMT3b) and mitochondrial transcription factor A (TFAM) as epigenetic markers. Additionally, we examined the expression patterns of mitomiRs related to the pathophysiology of CVD, including mitomiR24, mitomiR34a, mitomiR150, and mitomiR155, through real-time PCR amplification. Then, Ct values were recorded, and the fold change was calculated using the 2^-ΔΔCt method against an internal control. The post-translational epigenetic modifications of histone H3 protein, including methylation (mono-, di-, and tri-methylation of lysine residues at the 4th, 9th, 27th, 36th, and 79th positions), acetylation (at the 9th, 14th, and 18th positions of lysine), and phosphorylation (at the 10th and 28th positions of serine residues), were also assessed using a multimode ELISA (Enzyme-Linked Immunosorbent Assay) reader [22].

Evaluation of inflammatory cytokine and biological biomarker levels

To examine the inflammatory response, IL-6, TNF-α, and IFN-γ (interferon-gamma) levels, as well as CVD-specific biomarkers (NT-proBNP (N-terminal pro-B-type natriuretic peptide), myoglobin, and troponin I), were measured using Human GENLISA™ ELISA kits (KRISHGEN BioSystems, Cerritos, CA, USA). All the instructions from the protocol manual were strictly adhered to, and the readings were recorded using the Spark^®^ multimode microplate reader. The dynamic range of inflammatory cytokines was 7.81 to 500 pg/mL, and for NT-proBNP, myoglobin, and troponin I, it was 11.7, 0.059, and 0.116 pg/mL, respectively [23].

Measurement of mitochondrial electron chain complexes activity

MitoCheck Complex Activity Assay kits (Cayman, Ann Arbor, MI, USA; 700930 for complex I, 700940 for complex II, 700950 for complex III, 700990 for complex IV, and 701000 for complex V) were used to investigate age-related changes in mitochondrial activity. The isolated protein sample was obtained and processed following the assay methodology. The readings were recorded using the Spark^®^ multimode microplate reader [24].

Expression of telomerase reverse transcriptase (TERT)

The expression levels of the TERT gene were analyzed by amplification using a real-time PCR approach. Ct values were then recorded, and the fold change was calculated using the 2^-ΔΔCt method [25].

Estimation of genetic polymorphism in the GST P1 gene

The discontinuous genotypic traits were evaluated by conventional singleplex PCR amplification, followed by restriction digestion. The obtained amplicons were then analyzed using agarose gel electrophoresis, and the images were captured using a UV Gel Doc (Thermo Fisher Scientific). Furthermore, the frequency of alleles in the GST P1 gene genotype, along with the percentage risk factor, was calculated.

Correlation analysis

In this study, we employed the R programming language (R Foundation for Statistical Computing, Vienna, Austria) to analyze correlations among age and all parameters. Our focus was on uncovering insights with potential implications for biological and clinical contexts. The results from rigorous analysis were used as the dataset that formed the foundation for our subsequent analysis. Using Pearson correlation coefficients, calculated with R’s cor() function, we explored the strength and direction of relationships between these key variables. The resulting correlation matrix and scatterplot matrix provided a visual understanding of the associations. While acknowledging the limitations and assumptions inherent in correlation analyses, our study adhered to rigorous scientific standards.

The correlation coefficient, often denoted as r, measures the strength and direction of a linear relationship between two variables. The formula for the sample correlation coefficient, r, between variables X and Y is given by: \[ r = \frac{\sum (x_i - \bar{x})(y_i - \bar{y})}{\sqrt{\sum (x_i - \bar{x})^2 \cdot \sum (y_i - \bar{y})^2}} \]

CVD risk prediction utilizing machine learning models

Our study employed a structured machine learning pipeline to predict CVD risk using a dataset composed of key features such as age, proBNP levels, and mtDNA methylation. The data preprocessing phase involved handling missing values (using mean imputation for numerical and mode for categorical variables), encoding categorical variables through label encoding, and standardizing all numerical features using StandardScaler to ensure uniform scaling. The dataset was split into training (80%) and testing (20%) subsets for model development and evaluation. We trained four machine learning models - Logistic Regression, Decision Tree, Random Forest, and Support Vector Classifier (SVC) - on the processed training data. Hyperparameter tuning was performed using GridSearchCV with five-fold cross-validation to optimise model performance and generalizability. Model evaluation was conducted using multiple metrics, including cross-validation accuracy, confusion matrix, classification report (precision, recall, F1-score), and ROC-AUC (area under the receiver operating characteristic curve) scores, to capture both classification effectiveness and discrimination power. Among all models, the Random Forest classifier achieved the best overall performance and was selected as the final model. Feature importance was extracted from the trained Random Forest model to understand the relative contribution of each variable to CVD risk prediction. Additionally, explainability techniques such as LIME (Local Interpretable Model-Agnostic Explanations) and SHAP (SHapley Additive exPlanations) were used to provide both local and global interpretability of model predictions. Visualizations, including correlation heatmaps, pair plots, violin plots, and class-wise ROC curves, were employed to explore data relationships and validate the model’s interpretability.

The final Random Forest model was saved and integrated into a prediction pipeline, capable of classifying new patient inputs into one of three CVD risk levels: "Low," "Moderate," or "High." Preprocessing steps were embedded into the deployment pipeline to maintain consistency with the training process, ensuring reliable and explainable predictions in a real-world setting.

## Results

Subject demographics

Table 2 presents the comprehensive demographic data for the 259 subjects participating in the study, categorized into two distinct groups. Compared with healthy subjects, Group II exhibited significantly higher values across several health metrics, such as body mass index (BMI), waist circumference, blood pressure, cholesterol, and troponin I levels.

**Table 2:** Demographic data of Group I (Healthy) and Group II (Late) of cardiovascular risk Note: All variables present the mean ± standard deviation, or percent of total subjects with data for each variable. p-value was analyzed using the Chi-square test. BMI, body mass index

Variables	Group I (18-39 years) (N = 152)	Group II (40-80 years) (N = 105)
BMI (Kg/m^2^)	23.3 ± 3.28	28.04 ± 5.46
Age (years) (mean ± SD)	25 ± 5.34	57 ± 11
Waist circumference (cm)	86 ± 5	110 ± 12
Low blood pressure	(n = 4) 2.6%	(n = 71) 67.6%
High blood pressure	(n = 15) 9.86%	(n = 25) 23.8%
High cholesterol (mg/dL)	-	250 ± 5
Troponin I (pg/mL)	58.583 ± 13.9	98.769 ± 9.94
Diabetic	-	(n = 8) 7.6%
COVID-19 history	-	(n = 30) 28.57%

Mitochondrial oxidative DNA damage and ROS positively correlate with advancing age

The results revealed that the mean level of oxidative DNA damage was significantly higher in lymphocytic cells of Group II (older individuals) compared to Group I (younger individuals) (p < 0.0001), suggesting an age-associated increase in DNA damage. The elevated levels of DNA damage observed in the presence of FPG and glycosylase enzymes further support the role of ROS in oxidative stress-induced damage. Specifically, the mean FPG-modified DNA damage in Group II was 4.07 ± 0.7 ng/mL, significantly higher than in Group I (2.76 ± 0.8 ng/mL, p < 0.01).

**Figure 1:**
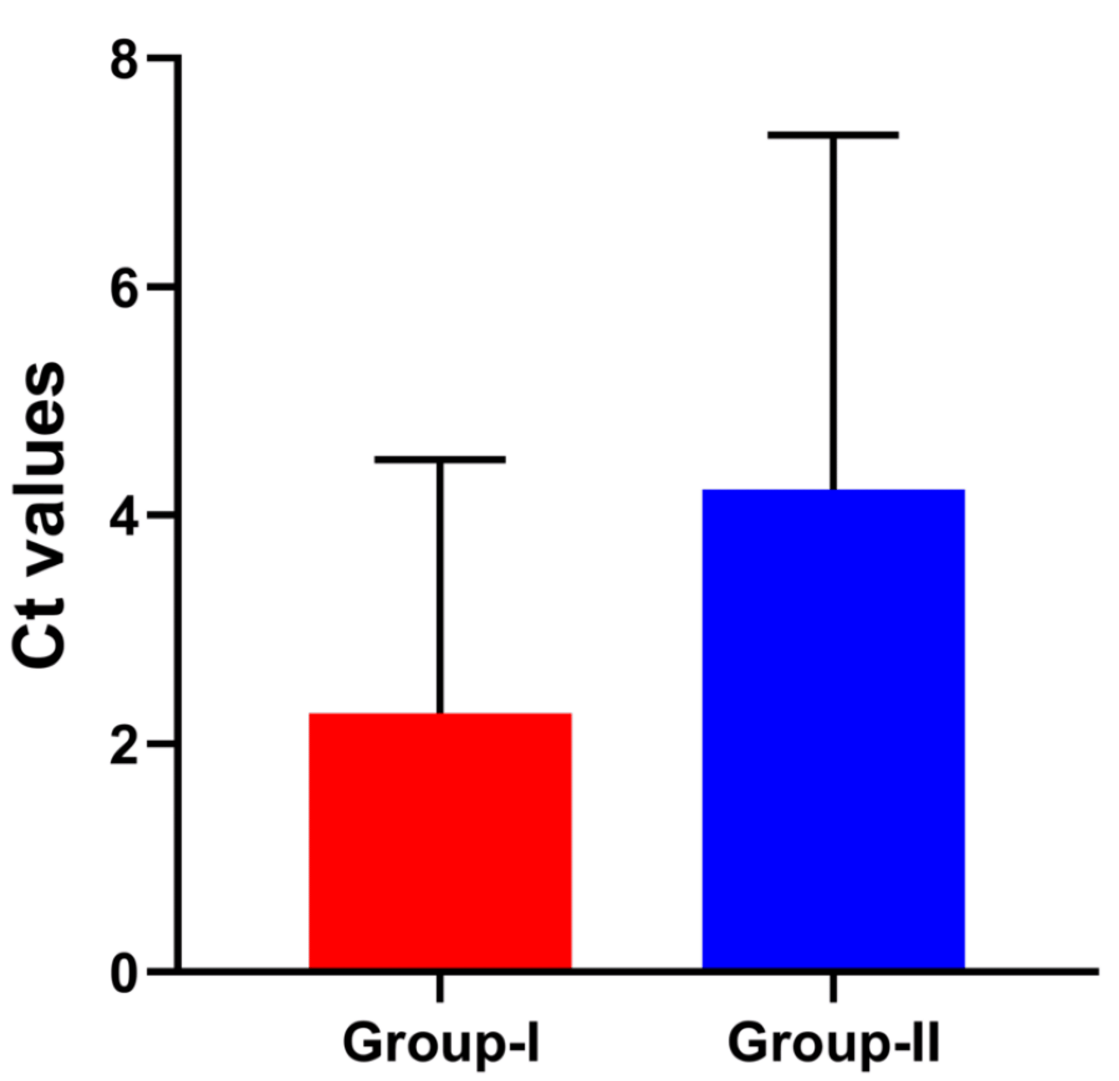
Bar graph showing comparative analysis of oxidative DNA damage estimated by FPG digestion method A bar graph showing a comparative analysis of oxidative DNA damage estimated by the FPG digestion method between older age individuals (Group II) and younger individuals (Group I), showing higher oxidative DNA damage and elevated reactive oxygen species (ROS) levels in Group II, indicating an association with advancing age. The y-axis represents ΔCt, calculated by subtracting the Ct value of enzyme-treated samples (Ct-T) from the Ct value of enzyme-non-treated samples (Ct-N). The values are expressed as mean ± SE, and p ≤ 0.05 was considered significant. FPG, Formamidopyrimidine DNA glycosylase

To further validate oxidative stress, intracellular ROS levels were assessed by flow cytometry using DCFH-DA staining. The control population (10.488%) represented untreated lymphocytic cells from healthy donors, establishing the baseline for ROS measurement. Compared to Group I, immunofluorescence analysis showed markedly elevated DCF (green) fluorescence in Group II, indicating higher ROS accumulation. DCF-positive cells in Group II averaged 37.540%, whereas Group I showed 18.383%, both significantly elevated compared to the control (p < 0.01) (Figure 1).

Analysis of mitochondrial epigenetic alterations associated with aging

As higher levels of ROS can impact mitochondrial homeostasis, we assessed various parameters to understand mitochondrial dynamics in relation to chronological age. The analysis began with the examination of fission and fusion genes. Our results revealed a significant r² = 0.6942 value for Group II, with a corresponding p-value <0.0001, suggesting upregulation in Group II individuals. While evaluating mitochondrial genes (MT-ATPase6, MT-ATPase8, MT-CO1, MT-ND6), we observed downregulated expression levels in Group II compared to Group I. The mean fold change values were 0.0059 ± 0.009, 0.0074 ± 0.006, 0.0092 ± 0.010, and 0.0025 ± 0.003 in Group II, with a significant r² value of 0.0829 and p = 0.0211, indicating altered biogenesis in Group II.

This altered mitochondrial functioning could profoundly result in activation of various stress response pathways and repair mechanisms. To check this, we also observed repair and ISR gene expression levels. The activity of repair genes (OGG1, APE, and POLG) was significantly different between Group II and Group I. The analysis revealed an r² = 0.8408 and a p < 0.0001, indicating significant alterations in the expression of these genes in Group II compared to Group I. Moreover, our results showed upregulated expression of ISR genes (OMA-1, DELE-1, and HRI) in relation to advancing age, as Group II individuals had a significant r² value of 0.9854 with a corresponding p-value <0.0001.

The induced oxidative stress can lead to mtDNA damage, which may subsequently alter the epigenetic machinery, resulting in hypo- or hyper-methylation. Our results demonstrated mtDNA methylation profiles in the D-loop, 12S, cytochrome B, and 16S rRNA regions. Group II exhibited mean fold change values calculated relative to the control group (Group I), which served as the baseline for comparison: 6.49 ± 0.86, 3.68 ± 0.49, 2.60 ± 0.13, 1.95 ± 0.49, and 1.02 ± 0.58, respectively, compared to Group I with mean fold change values of 4.56 ± 1.11, 0.72 ± 0.64, 1.33 ± 0.17, 1.10 ± 0.63, and 3.13 ± 0.74. The correlation of these values with age was assessed, revealing an r² = 0.5205 and p < 0.0001, indicating significant associations between mtDNA methylation and aging. Further, the methylation is regulated by a set of enzymes, DNMTs (DNMT1, DNMT3a, and DNMT3b), whose activity profile in Group II was 8.17 ± 0.12, 6.41 ± 0.09, and 5.99 ± 0.13, respectively. Particularly, DNMT1 and DNMT3A regions showing increased DNMT expression were generally associated with hypomethylation.

**Figure 2:**
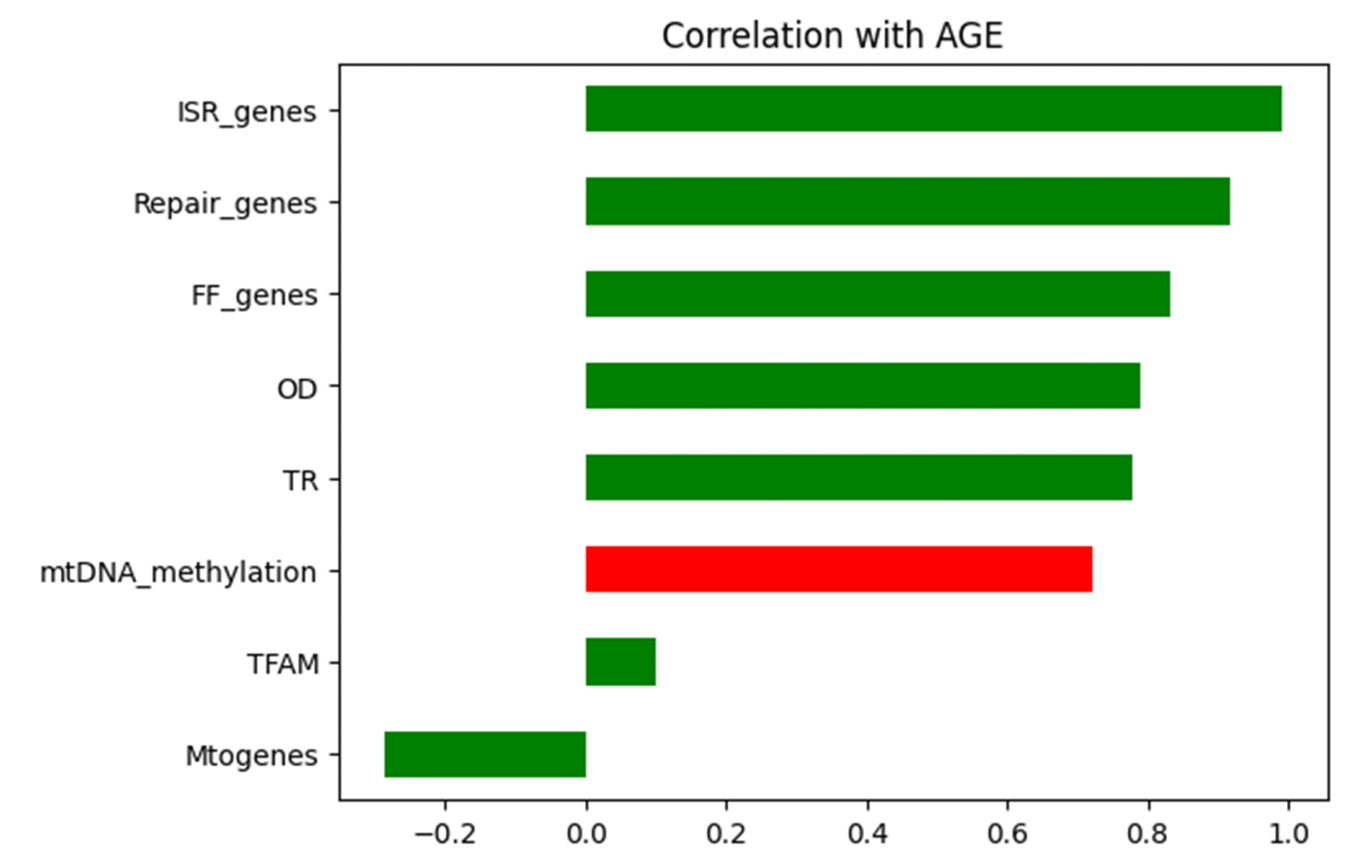
Horizontal bar graph representing the direct correlation of key parameters of mitochondrial dynamics A horizontal bar graph representing the direct correlation of key parameters of mitochondrial dynamics with age, showing modified levels of each parameter when assessed with advancing age. The red bar indicates mitochondrial DNA (mtDNA) methylation, which is the primary focus of the study and has therefore been differentiated. The correlations between these parameters are also shown, reflecting how changes in one parameter can influence others, leading to altered mitochondrial homeostasis. Modified levels of each parameter are represented, highlighting significant trends with advancing age. Correlation coefficients and statistical significance are indicated where applicable, with p-values <0.05.

**Figure 3:**
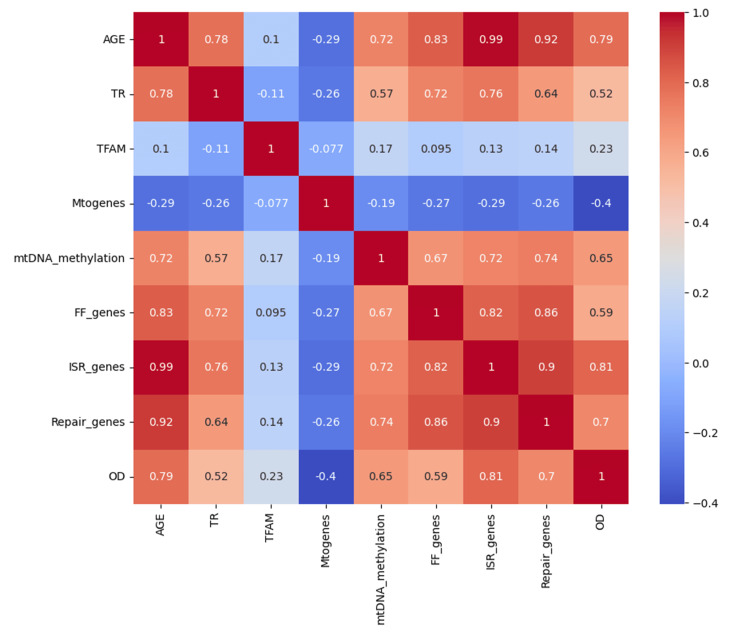
Heatplot illustrating the relationship between mitochondrial integrity parameters A heatplot illustrating the relationship between mitochondrial integrity parameters and age, with key parameters including biogenesis, fusion, fission, repair, integrated stress response (ISR), and oxidative stress levels. The color gradient in the heatplot indicates the intensity of changes, with warmer colors signifying greater alterations. Significant alterations are highlighted by the color intensity, with statistical significance assessed at p-values <0.05.

Cox regression analysis model was performed to examine mitoepigenetic alterations associated with advancing age. Using a backward elimination approach, two variables - mitoepigenetic alterations and aging - were retained in the model. This analysis revealed how key parameters, crucial for maintaining mitochondrial homeostasis, correlate with aging. We assessed how mitochondrial epigenetics change with age and found that certain parameters, such as mtDNA methylation, fusion-fission genes, ISR, repair genes, and oxidative DNA damage, exhibited significant alterations as age increases (Figures 2-3).

**Figure 4:**
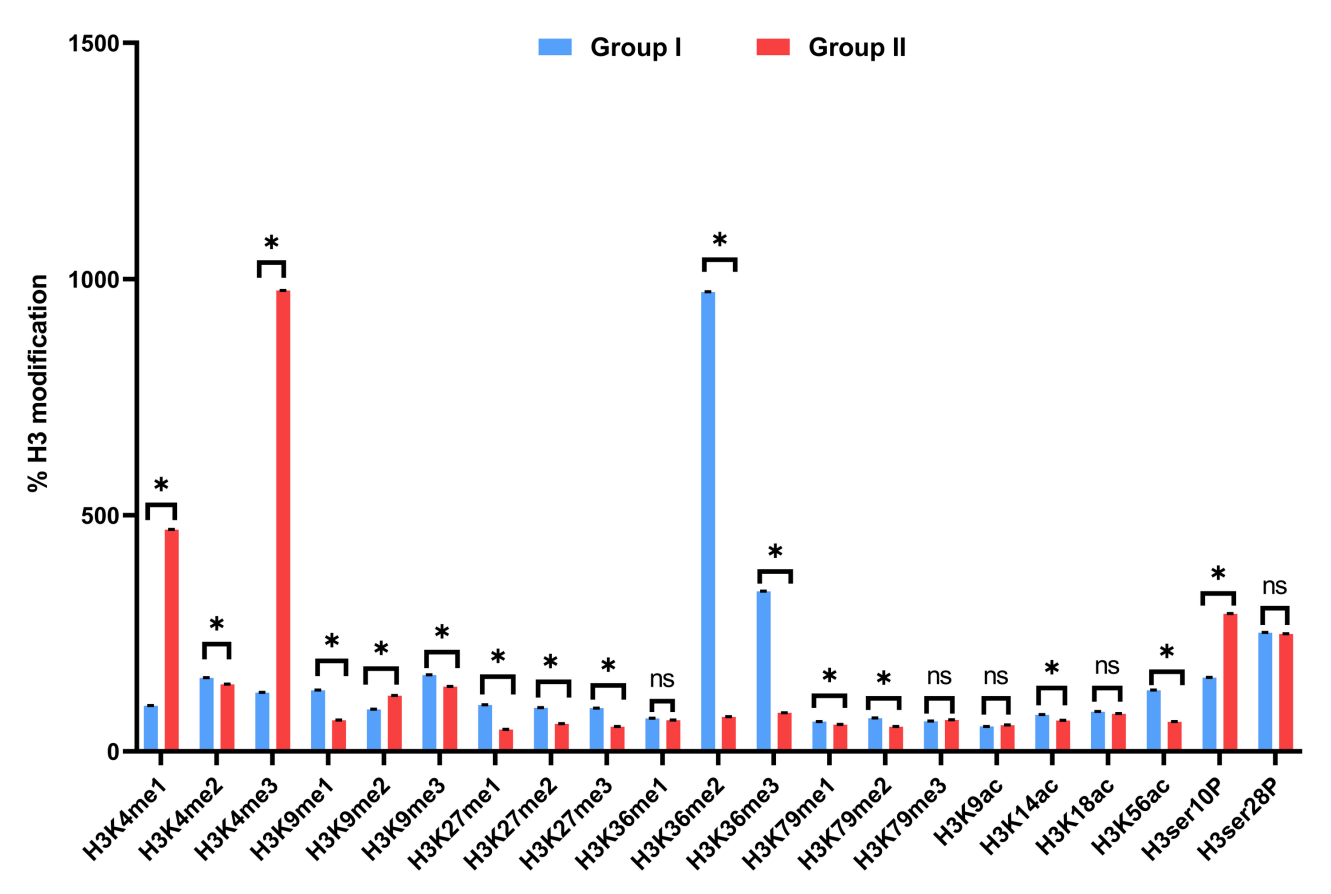
Histogram showing the analysis of 21 histone H3 modifications in Group I and Group II The X-axis represents individual histone H3 modifications, while the Y-axis depicts % H3 modifications. Values are expressed as mean ± SE. Differences between groups are indicated by statistical significance: NS represents non-significant differences, while an asterisk (*) denotes significance with p ≤ 0.05. The graph highlights the comparative levels of histone H3 modifications between the two groups, with significant differences marked accordingly.

We also analyzed the expression patterns of mitomiRs, which play crucial regulatory roles, and showed that Group II (3.29 ± 0.88, 1.09 ± 0.16, 2.66 ± 0.83, and 2.21 ± 0.90) exhibited significantly higher fold change compared to Group I. This indicates that mitomiRs in Group II are more highly expressed, reflecting their increased regulatory influence, as confirmed by the changes in expression levels and the band intensities observed after agarose gel electrophoresis. Histones also bear key regulatory functions that are associated with aging processes. Our results demonstrated histone methylation, acetylation, and phosphorylation patterns in Group II in comparison to Group I, with p ≤ 0.05 (Figure 4).

Assessment of age-associated variations in inflammatory cytokines

**Figure 5:**
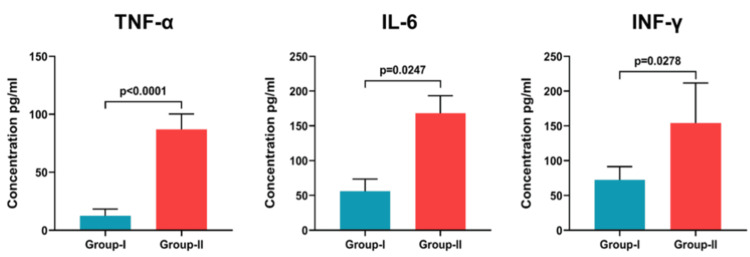
Graph depicting the levels of pro-inflammatory cytokines in cells exposed to different concentrations in Group I and Group II The X-axis represents concentrations of the treatment in Group I and Group II, while the Y-axis depicts levels of pro-inflammatory cytokines, expressed as mean ± SE. The graph highlights variations in cytokine levels across different concentrations and between the two groups, with significant differences in Group II marked by their correlation with age. TNF-α, tumor necrosis factor-alpha; IL-6, interleukin-6; IFN-γ, interferon-gamma

Our analysis revealed a significant increase in pro-inflammatory cytokines in older individuals (Group II), concomitant with altered mitochondrial redox homeostasis. Compared to younger controls, Group II exhibited markedly higher levels of IL-6 (p = 0.0247), TNF-α (p < 0.0001), and IFN-γ (p = 0.0278), demonstrating a progressive inflammatory shift with advancing age. While these findings highlight a strong correlation between mitochondrial dysfunction and inflammation, we acknowledge that additional variables, such as comorbidities, medication use, and metabolic health, could influence cytokine expression. To mitigate confounding effects, our study excluded participants with chronic inflammatory conditions, recent infections, or immunosuppressive therapies. The observed cytokine elevation aligns with established mechanisms linking oxidative stress to NLRP3 inflammasome activation, supporting the role of mitochondrial redox imbalance in age-related systemic inflammation (Figure 5).

Measurement of mitochondrial electron chain complexes activity

To evaluate mitochondrial functional alterations associated with aging, the specific activities of electron transport chain (ETC) complexes I to V were quantified in lymphocytic cells from both age groups. Group II (older individuals) demonstrated slightly higher mean specific activities for all five complexes compared to Group I (younger individuals). The enzyme activities in Group II were recorded as follows: complex I, 47.05 ± 2.11; complex II, 38.16 ± 11.90; complex III, 27.63 ± 5.16; complex IV, 73.90 ± 3.07; and complex V, 67.65 ± 12.17. Correspondingly, Group I showed lower values: complex I, 42.79 ± 2.85; complex II, 34.59 ± 2.90; complex III, 21.44 ± 4.36; complex IV, 65.65 ± 4.45; and complex V, 58.53 ± 18.20.

**Figure 6:**
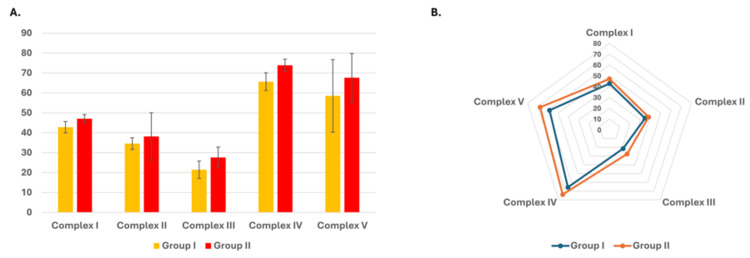
Specific activities of electron transport chain (ETC) complex enzymes in Group I and Group II (A) Bar graph comparing mean specific activities (units/mg protein) of ETC complexes (I-V) between Group I and Group II, with Group II exhibiting higher overall enzyme activities compared to Group I. (B) Segmented representation of relative enzyme-specific activities for selected complexes (I, II, III, and IV), further emphasizing the between-group differences. Data are presented as mean values.

All enzyme activities were normalized to total protein content. Although no statistical significance testing was conducted, these modest elevations in ETC activity in older individuals may reflect early compensatory mitochondrial responses to age-associated metabolic demands or oxidative stress. These findings warrant further validation in larger, statistically powered studies (Figure 6).

Estimation of genetic polymorphism in the GST P1 gene

**Figure 7:**
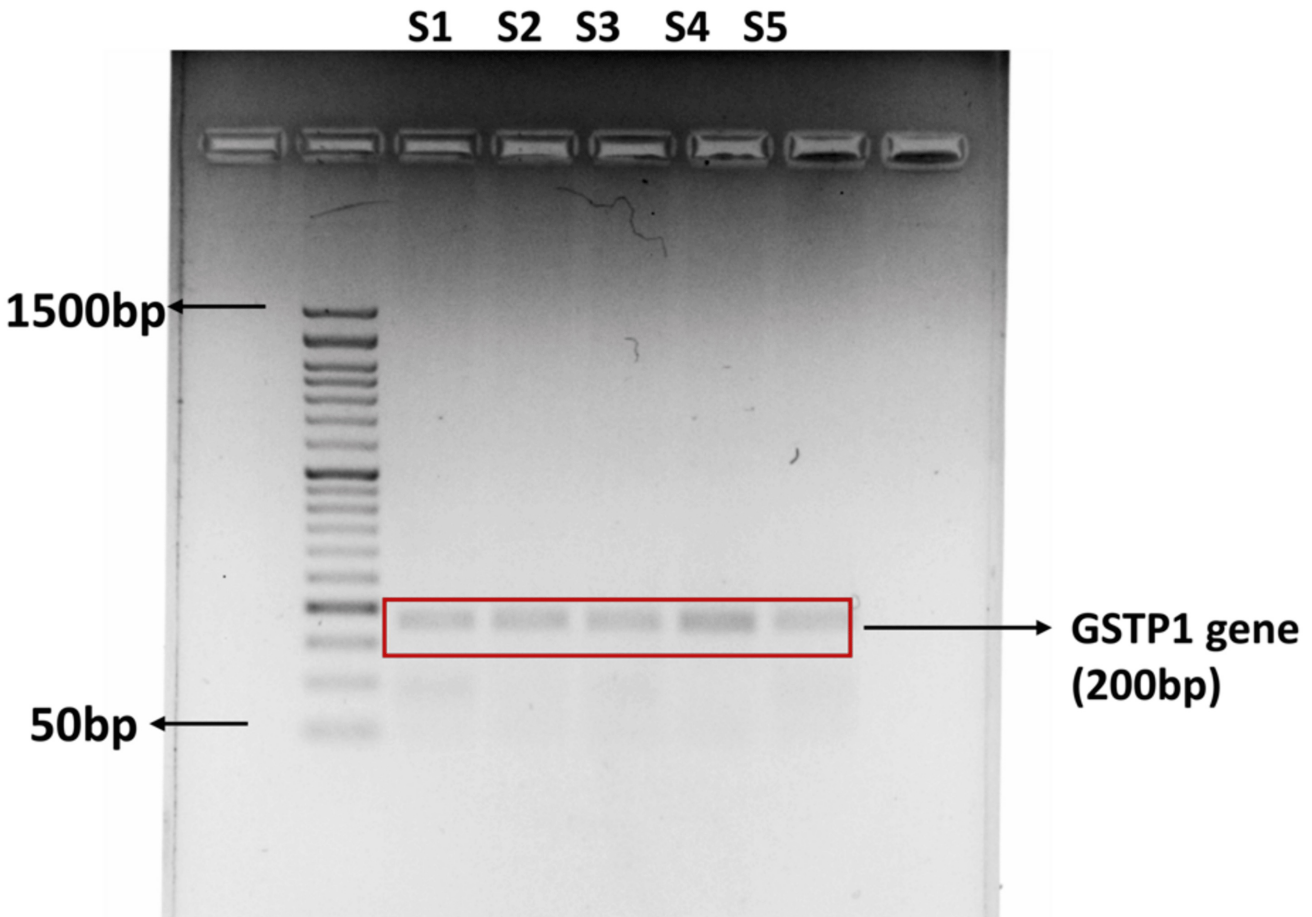
Gel electrophoresis image illustrates genetic polymorphism analysis in the GSTP1 gene Each lane represents an amplified DNA sample, with visible bands corresponding to specific allelic variants of the GSTP1 gene.

We assessed ETC complex enzyme activity (complexes I-V) and found slight differences in activity profiles between the two groups. Group II exhibited mean specific activities of 47.05 ± 2.11, 38.16 ± 11.90, 27.63 ± 5.16, 73.90 ± 3.07, and 67.65 ± 12.17, whereas Group I showed slightly lower values of 42.79 ± 2.85, 34.59 ± 2.90, 21.44 ± 4.36, 65.65 ± 4.45, and 58.53 ± 18.20, respectively (Figure 7).

Multivariate correlation analysis

**Figure 8:**
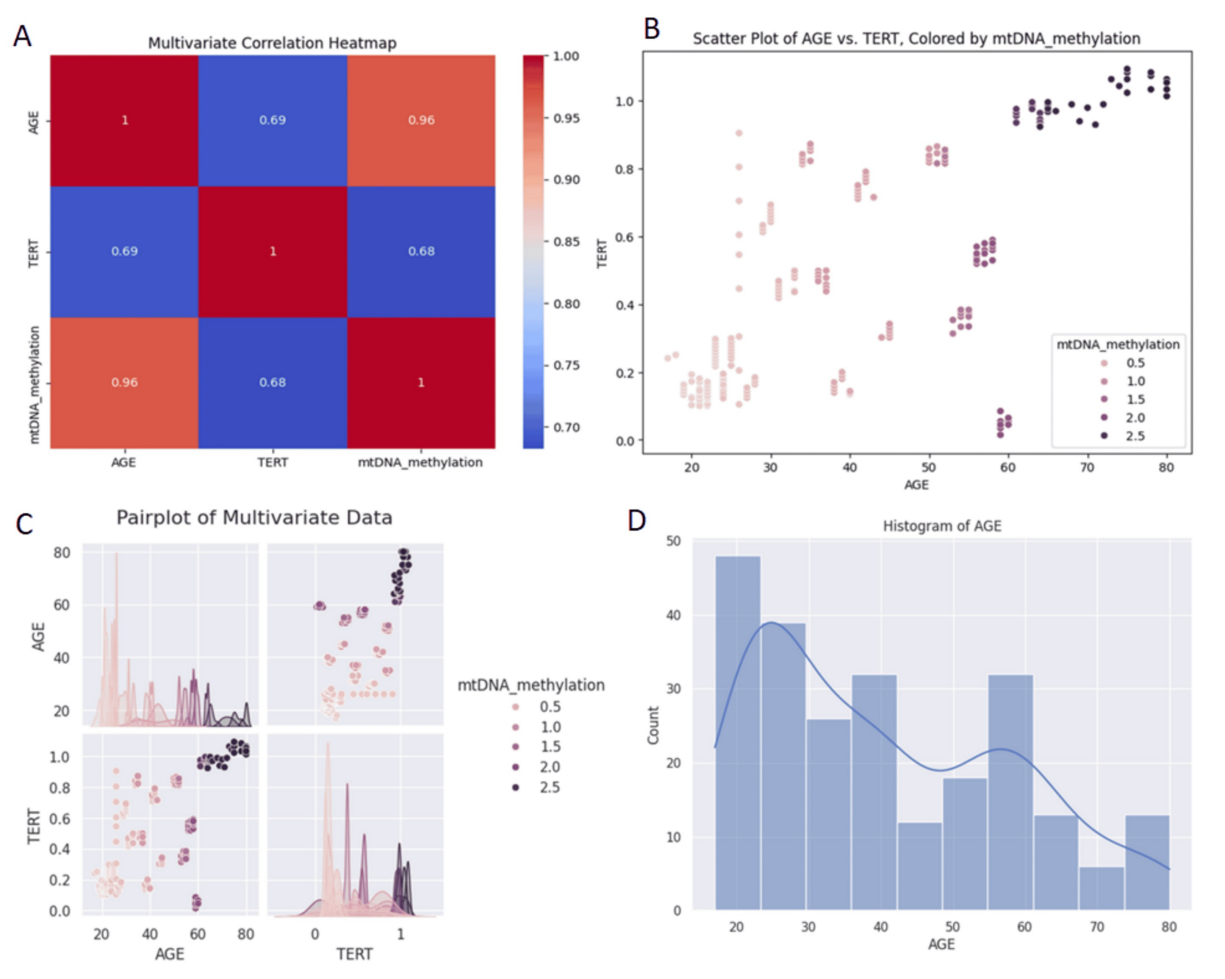
Multivariate cox regression analysis, comprising four models each represents key associations with other covariates Model A: A multivariate correlation heatmap between age, mitochondrial DNA (mtDNA) methylation, and telomerase reverse transcriptase (TERT). The X-axis and Y-axis showcase the variables (age, mtDNA methylation, and TERT expression). The heatmap displays the correlation coefficients between each pair of variables, with color intensity indicating the strength and direction of the correlation; warmer tones depict higher intensity or a positive correlation. Model B: A scatter plot showing the association between TERT expression and aging, with mtDNA methylation levels. Each point represents an individual sample, and the plot depicts a parallel association between TERT expression, aging, and mtDNA methylation. Model C: A pair plot of multivariate data revealing covariate balance among aging, TERT expression, and mtDNA methylation. The diagonal panels show histograms of each variable, while the off-diagonal panels display scatter plots of pairwise relationships between the variables. The plot illustrates a positive correlation between mtDNA methylation and chronological age, as shown by the upward trend in the scatter plot. In contrast, TERT expression demonstrates a negative correlation with age, reflected in the downward trend in its scatter plot. Statistical significance of the correlations is denoted where applicable, with p-values < 0.05. Model D: A histogram depicting the distribution of different aging cohorts within the multivariate dataset. X-axis: Aging cohorts or age groups. Y-axis: Frequency or count of samples within each aging cohort. Each bar represents the number of samples that fall into a specific aging cohort, showing the distribution of the sample population across different age groups.

After assessing all variables, we performed multivariate Cox regression analysis to explore the relationships between mtDNA methylation, TERT expression, CVD risk, and age. In Figure 8, Model A (visualized through a multivariate correlation heatmap), we adjusted for covariates including age, mtDNA methylation, and TERT. This analysis revealed a significant negative correlation between TERT and age (r² = 0.6070, p < 0.0001), and a positive correlation between mtDNA methylation and age (r² = 0.5205, p < 0.0001), as shown in Figure 7. In Model B (depicted as a scatter plot), we observed a linear association between mtDNA methylation and both TERT expression and age, with a moderate correlation (r² = 0.3218, p < 0.0001). Model C (represented by a pair plot) illustrated the covariate balance among age, TERT, and mtDNA methylation, showing consistent trends: mtDNA methylation increased with age, while TERT decreased. Finally, Model D (a histogram) displayed the distribution of aging cohorts within the multivariate dataset. It is important to note that, while these associations were statistically significant, they represent correlations, not causative relationships.

**Figure 9:**
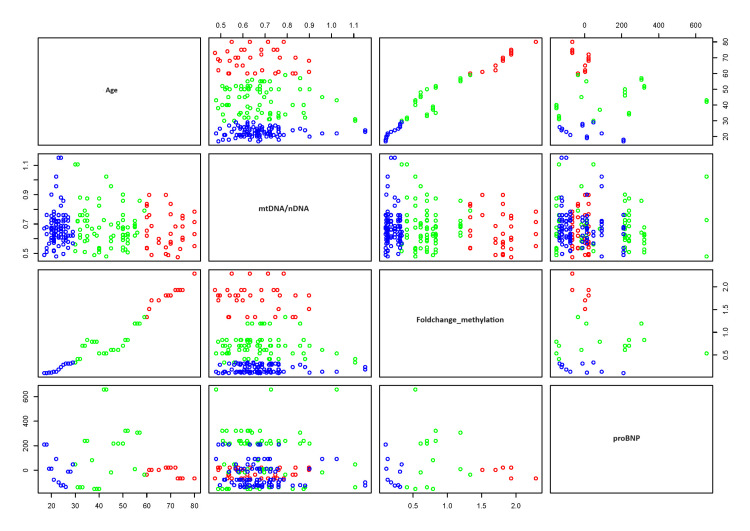
Scatter plot evaluating cardiovascular disease (CVD) risk with advancing age, focusing on mtDNA methylation, mtDNA copy number, and proBNP levels The plot reveals a weak negative correlation between age and the mtDNA/nDNA ratio, indicating a slight decline in mtDNA copy number relative to nuclear DNA with age. Conversely, a strong positive correlation is observed between age and fold change in methylation, reflecting increased mtDNA methylation with advancing age. Additionally, a weak positive correlation is seen between age and proBNP levels, suggesting a modest rise in proBNP as individuals age. Statistical significance is denoted where applicable, with p-values < 0.05. mtDNA/nDNA, mitochondrial DNA/nuclear DNA; proBNP, pro-B-type natriuretic peptide

Furthermore, to evaluate CVD risk in advancing age with respect to mtDNA methylation, we observed associations with mtDNAcn, proBNP, and the methylation status of CpG sites. Our results show a weak negative correlation between age and the mtDNA/nDNA (nuclear DNA) ratio, indicating a slight decline in mtDNAcn relative to nDNA with age. Conversely, there is a strong positive correlation between age and fold change methylation, reflecting increased mtDNA methylation with advancing age. Additionally, there is a weakly positive correlation between age and proBNP, indicating a modest rise in proBNP levels as individuals age (Figure 9 and Table 3).

**Table 3:** Pearson correlation coefficients illustrating the relationships between age and various biomarkers The table provides the strength and direction of associations between age and the following variables. mtDNA/nDNA, mitochondrial DNA/nuclear DNA; proBNP, pro-B-type natriuretic peptide

Pearson coefficient correlation				
Variables	Age	mtDNA/nDNA	Fold change methylation	proBNP
Age	1	-0.07193	0.962354	0.180244
mtDNA/nDNA	-0.07193	1	-0.08207	-0.02361
Fold change methylation	0.962354	-0.08207	1	0.078779
proBNP	0.180244	-0.02361	0.078779	1

AI prediction model analysis for CVD

**Figure 10:**
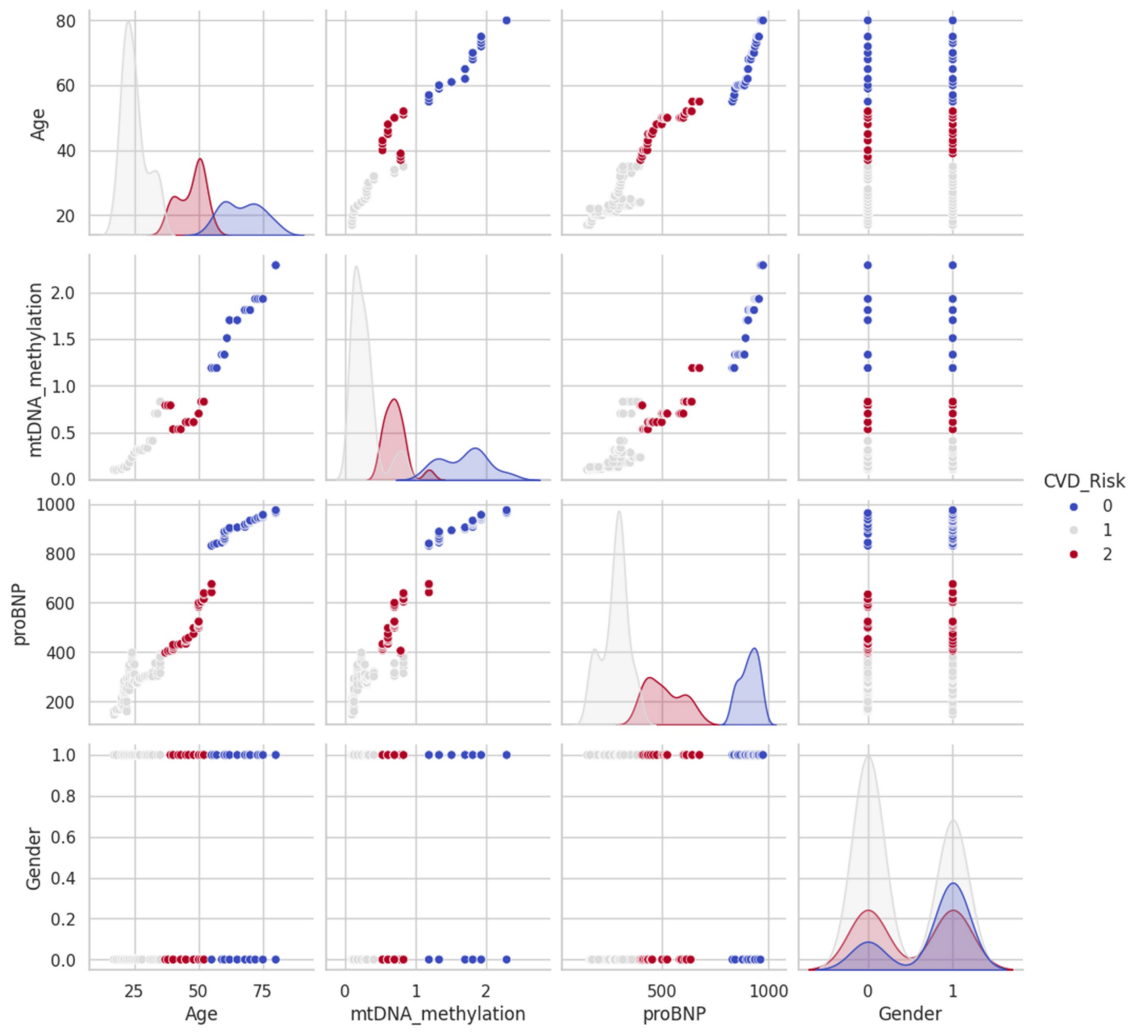
Featuring a grid of scatter plots and histograms, this analysis compares various variables such as age, mtDNA methylation, proBNP levels (a biomarker for heart failure), and gender The scatter plots show the relationships between pairs of variables, while the histograms on the diagonal illustrate the distribution of each individual variable. The data points in the scatter plots are color-coded to indicate different levels of cardiovascular disease (CVD) risk, categorized as 0 (High Risk), 1 (Low Risk), and 2 (Moderate Risk). mtDNA, mitochondrial DNA; proBNP, pro-B-type natriuretic peptide

The dataset analyzed in this study comprised 259 samples, categorized into three CVD risk levels: high (0), moderate (2), and low (1). An initial exploratory data analysis revealed several notable relationships among the features. A moderate positive correlation was observed between age and mtDNA methylation, suggesting that mitochondrial epigenetic modifications may accumulate with age. Additionally, proBNP levels exhibited a stronger positive correlation with age, consistent with age-related increases in cardiac stress and ventricular dysfunction. Although the correlation between mtDNA methylation and proBNP levels was weaker, it still points to a potential interplay, possibly mediated by age or underlying cardiovascular changes. However, due to the relatively small sample size (n = 259), the R² values from regression analyses may underestimate the true strength of these associations, as smaller datasets are more prone to statistical noise and reduced generalizability (Figure 10).

**Figure 11:**
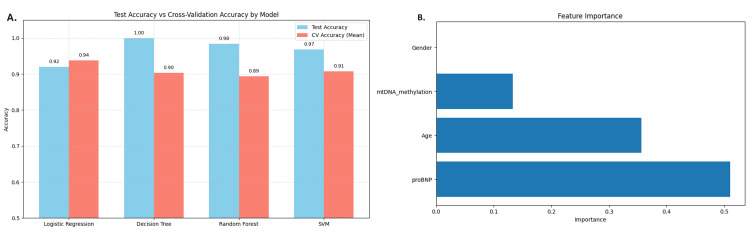
Bar chart compares the accuracy of the models (A) The bar chart compares the accuracy of four models, along with the Mean Cross-Validation Accuracy: Logistic Regression, Decision Tree, Random Forest, and SVM. (B) The horizontal bar chart, titled “Feature Importance,” illustrates the significance of four features for the Random Forest model: Gender, mtDNA_methylation, Age, and proBNP. mtDNA, mitochondrial DNA; proBNP, pro-B-type natriuretic peptide

**Figure 12:**
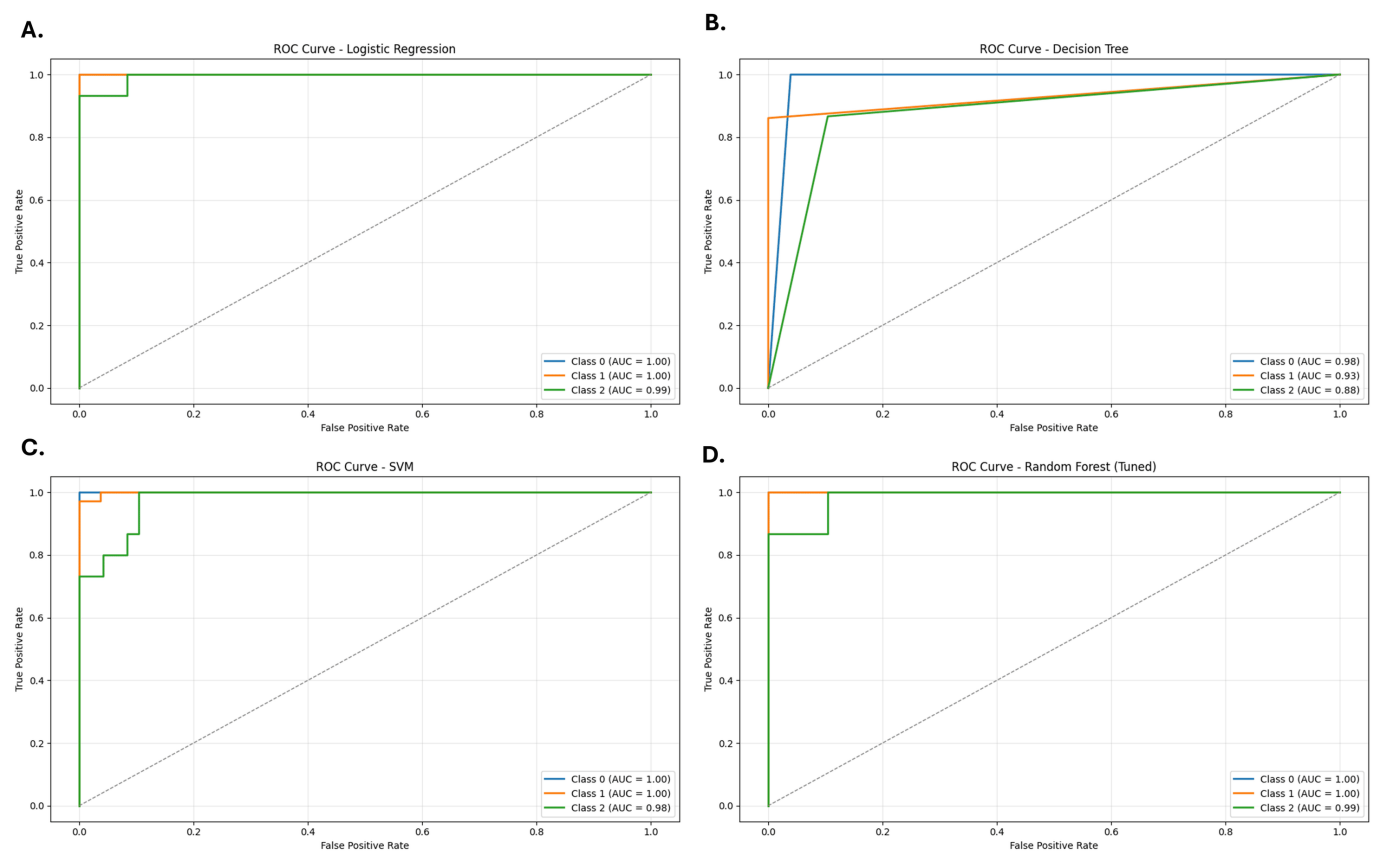
Receiver operating characteristic (ROC) curves for multiclass classification of cardiovascular disease (CVD) risk using four machine learning models Note: Class 0 = High Risk, Class 1 = Low Risk, Class 2 = Moderate Risk. All ROC curves were generated using a one-vs-rest strategy under stratified five-fold cross-validation. (A) Logistic Regression achieved perfect discrimination across all three classes, with an area under the curve (AUC) of 1.00 for Class 0 (High Risk), Class 1 (Low Risk), and Class 2 (Moderate Risk). (B) The Decision Tree model showed strong performance, with AUC values of 0.98 (Class 0), 0.93 (Class 1), and 0.88 (Class 2), though slightly lower than the other models. (C) The Support Vector Machine (SVM) demonstrated near-perfect performance, yielding AUCs of 1.00 (Class 0), 1.00 (Class 1), and 0.98 (Class 2). (D) The tuned Random Forest exhibited consistently excellent results, with AUCs of 1.00 (Class 0), 1.00 (Class 1), and 0.99 (Class 2), confirming its robustness and suitability for CVD risk classification.

To ensure robust CVD risk prediction, four machine learning models - Logistic Regression, Decision Tree, Random Forest, and Support Vector Machine (SVM) - were developed and evaluated. All models were trained using stratified five-fold cross-validation to mitigate overfitting and assess performance on unseen data (Table 4). Although some models approached high accuracy levels (0.95-1.00) (Figure 11), raising potential overfitting concerns, this was addressed using additional performance metrics, including precision, recall, F1-score, and ROC-AUC (Figure 12). Among all models, Random Forest consistently outperformed the others across these metrics and was therefore selected for further interpretability and visualization.

**Table 4:** Performance metrics and cross-validation results for cardiovascular disease (CVD) risk prediction models Note: The values for Precision, Recall, and F1-Score represent macro averages across the three CVD risk classes (Low, Moderate, and High). Cross-validation results reflect stratified five-fold CV on the training data, with standard deviation (SD).

Model	Accuracy	Precision	Recall	F1-score	Cross-validation accuracy
Logistic Regression	0.921	0.93	0.89	0.89	0.938
Decision Tree	1.000	1.00	1.00	1.00	0.903
Random Forest	0.984	0.99	0.98	0.98	0.893
Support Vector Machine	0.968	0.98	0.96	0.97	0.908

**Figure 13:**
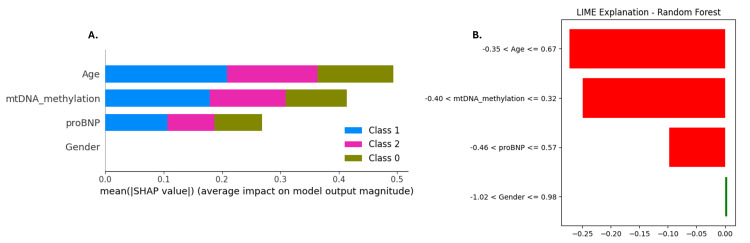
Interpretability of Random Forest model using SHAP and LIME (A) SHAP summary plot showing average feature importance across all cardiovascular disease (CVD) risk classes. proBNP, mtDNA methylation, and age were the top contributors, while gender had minimal impact. (B) LIME explanation for a high-risk prediction, highlighting that lower values of age, mtDNA methylation, and proBNP reduced the model’s confidence in high-risk classification. SHAP provides global interpretability, while LIME offers local interpretability. LIME, Local Interpretable Model-Agnostic Explanations; SHAP, SHapley Additive exPlanations; mtDNA, mitochondrial DNA; proBNP, pro-B-type natriuretic peptide

Feature importance derived from the Random Forest model indicated that proBNP was the most influential predictor, followed by age and mtDNA methylation. Gender, encoded as a binary variable (0 for female and 1 for male), had the least influence. To account for potential feature interdependence and nonlinearity, SHAP values were computed, providing a more nuanced and interpretable understanding of the model’s decision-making process. SHAP analysis confirmed the dominance of proBNP in driving model predictions and revealed non-linear interactions among age and mtDNA methylation, indicating that these features influence CVD risk in a context-dependent manner (Figure 13A). To complement the global interpretability offered by SHAP, LIME was employed to examine specific, individual-level predictions. For a representative high-risk instance, LIME revealed that low values of age, mtDNA methylation, and proBNP levels all negatively contributed to the classification - reinforcing that the model relies on elevated values of these features to assign high-risk scores. The LIME explanation was consistent with SHAP insights and highlighted the trustworthiness of the model’s logic at the individual level. Importantly, the alignment between SHAP and LIME interpretations supports the credibility of the Random Forest model’s decisions, suggesting that predictions are rooted in biologically plausible, data-driven patterns rather than artifacts or spurious correlations (Figure 13B).

**Figure 14:**
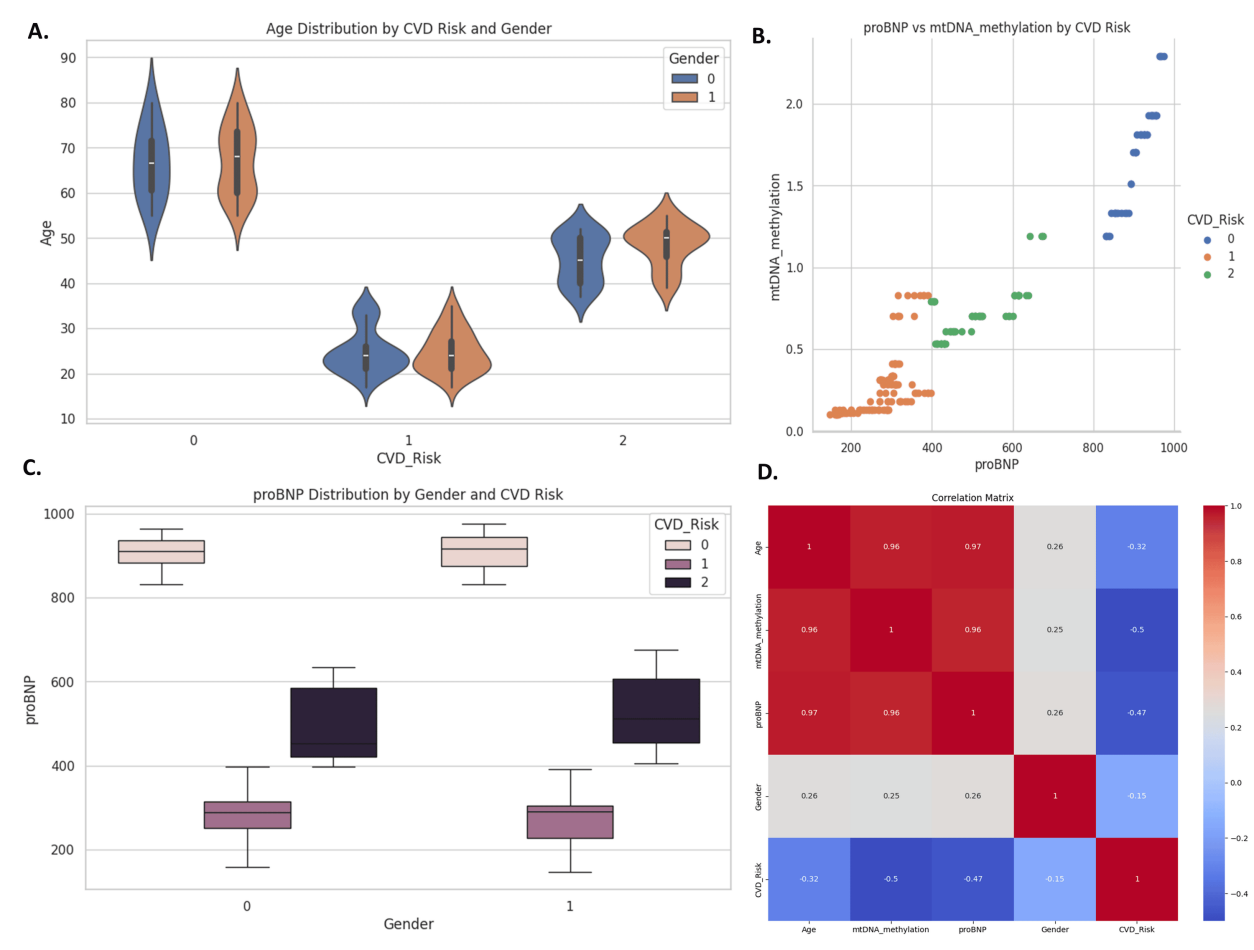
Key visualizations of cardiovascular disease (CVD) risk (0: High Risk, 1: Low Risk, 2: Moderate Risk) and related features Panel A shows a violin plot, where older individuals - especially males - are more likely to be in the high-risk group. Panel B illustrates a scatter plot, linking higher proBNP levels and mtDNA methylation with increased cardiovascular disease (CVD) risk. Panel C uses box plots to show that males with elevated proBNP levels are at greater risk. Panel D is a correlation matrix, highlighting strong associations between age, mtDNA methylation, proBNP, and CVD risk, reinforcing their role as critical predictors. mtDNA, mitochondrial DNA; proBNP, pro-B-type natriuretic peptide

Visualization of model predictions further reinforced these findings. Violin and box plots demonstrated that individuals classified in the high-risk group tended to have elevated proBNP levels and older age, with males more frequently appearing in this group. A scatter plot of mtDNA methylation versus proBNP levels revealed clustering among high-risk individuals, underlining their joint predictive value (Figure 14).

The results support the feasibility of using a machine-learning framework, incorporating age, mtDNA methylation, and proBNP, to predict CVD risk. At the same time, the study highlights important limitations, including the modest sample size, potential feature collinearity, and the need for further sex-stratified and prospective validation to improve generalizability and clinical translation.

## Discussion

In the present study, we elucidated the alterations in mitochondrial epigenetics with increasing age and their role in the increased susceptibility to age-related cardiovascular risks by conducting a longitudinal cohort study in India, categorizing two age groups: Group I (18-38 years, younger-aged individuals) and Group II (39-65 years, older-aged individuals). We observed variations in mitoepigenetic profiling of Group II that point to changes in mitochondrial dynamics associated with aging, posing a vulnerability to cardiovascular health. Further, we hypothesized the potential of mtDNA methylation as a potential predictive biomarker for age-associated CVD. Our initial observation revealed elevated ROS levels in older individuals, as detected by comparative flow cytometric profiling, indicating uncontrolled ROS with age. According to the free radical theory of aging, cellular damage arises from ROS, with mitochondria serving as both a major source and target, acting as a potential "aging clock" [26]. This finding aligns with research showing that aged individuals experience increased mitochondrial ROS, which triggers protective mechanisms [27].

Age-associated functional decline in the mammalian heart is closely tied to increased oxidative stress, largely driven by dysregulated mitochondrial metabolism and elevated production of ROS. Preserving functional capacity, such as exercise tolerance and cardiac output, should be a primary therapeutic goal in older adults with CVD, noting that oxidative stress and mitochondrial dysfunction are major contributors to this decline [28]. Emelyanova et al. (2016) demonstrated that in human atrial tissue, particularly from patients with atrial fibrillation, there is selective downregulation of mitochondrial ETC activity, accompanied by elevated ROS levels. The reduction in ETC complex I and complex IV activities leads to inefficient electron transfer and electron leakage, contributing to mitochondrial-derived ROS accumulation. These perturbations in mitochondrial bioenergetics and redox homeostasis are hallmarks of both pathological cardiac remodeling and cardiac aging with advancing age [29,30]. Our findings of higher oxidative damage in older individuals further support this, including increased mtDNA damage and reduced mtDNAcn. This aligns with the work of Cochemé et al. (2011), who used a ratiometric mass spectrometry probe to show that H₂O₂ levels increase within the mitochondrial matrix during aging in *Drosophila*, providing direct evidence of mitochondrial ROS accumulation over time, which drives impaired mitophagy and reduced biogenesis, collectively contributing to age-related functional decline [31,32], and is further associated with aging-related CVDs [33].

The presence of higher 8-oxo-dG, a marker of oxidative DNA damage, indicates compromised mtDNA integrity and suggests that oxidative stress is a significant factor in mitochondrial dysfunction. Notably, increased 8-oxo-dG levels can impair mitochondrial function and enhance ROS production, creating a feedback loop that leads to even greater oxidative damage and further accumulation of 8-oxo-dG. Several findings indicated a noticeable tendency toward increased oxidative DNA damage, including single-strand breaks in elderly males and during depressive episodes in older adults [34,35]. Consequently, elevated ROS contributes to accelerated aging and diminished quality of life in the elderly. The cumulative oxidative damage from elevated ROS impairs cellular function, leading to cellular senescence and exacerbating the overall decline associated with aging.

Our study revealed notable changes in mitochondrial dynamics, highlighting the impact of these epigenetic modifications on mitochondrial function and aging. Our data showed upregulation of key regulators of mitochondrial homeostasis, i.e., fusion (MFN1, MFN2, and OPA1) and fission (Drp1, Fis1, and MFF) genes, in older individuals, suggesting an imbalance associated with aging. Increased fission, despite the presence of fusion, overwhelms the system and leads to a net increase in the number of smaller, fragmented mitochondria. This is supported by the observed reduction in mtDNAcn, which is commonly associated with fragmented mitochondrial networks and impaired biogenesis. This happens because fragmented mitochondria may have more difficulty replicating their DNA and become more susceptible to mitophagy. Although mitochondrial fission and fusion are crucial for maintaining mitochondrial health - as fusion supports network formation, while fission causes fragmentation and functional impairment - their roles in aging remain complex and not fully understood. Previous research has shown that mitochondrial networks become fragmented and disorganized with age or in diseased models, typically attributed to increased fission [36,37]. Conversely, swollen mitochondrial aggregates are believed to result from excessive fusion and impaired mitophagy [38]. Despite the observed fragmentation, fission proteins like DRP1 and FIS1 are often downregulated in aged cells [39-41]. Additionally, PGC-1α, a key regulator of mitochondrial biogenesis and function, influences mitochondrial dynamics by promoting the expression of both fusion and fission proteins. However, its role can be altered with age, potentially contributing to the observed imbalance, as supported by existing literature on PGC-1α's role in regulating mitochondrial biogenesis [42]. In mammalian cells, overexpression of mitochondrial fusion proteins MFN1 and MFN2 can lead to either clustering of spherical mitochondria or their elongation [43], further illustrating the intricate balance required for proper mitochondrial function and the complex role PGC-1α may play in maintaining this balance. Contrary to our observations, previous findings revealed that promoting a shift in mitochondrial dynamics toward increased fission and decreased fusion in middle-aged animals prolongs organismal health and lifespan, mainly due to activation of various cellular pathways [44]. Taken altogether, any imbalance in biogenesis could lead to significant alterations that may contribute to age-related diseases.

Another observation made was the downregulation of mitochondrial genes (MT-ATPase6, MT-ATPase8, MT-CO1, and MT-ND6) in the aged group, which was accompanied by changes in the activity of mitochondrial respiratory chain complexes (ETC). Mitochondrial metabolism is at the core of a network of cellular processes linked to aging [45,46]. Building on our observations, multiple studies have shown that aging results in uneven declines across mitochondrial ETC complexes. For example, Tatarková et al. (2011) reported differential reductions in the activities of ETC complexes in aged rat hearts, along with increased oxidative damage. Similarly, Ferguson et al. (2005) demonstrated an age-dependent decline in mitochondrial respiration and ETC efficiency in *Drosophila melanogaster*, further supporting the notion that mitochondrial dysfunction during aging is complex-specific and evolutionarily conserved [47,48].

This oxidative damage can affect the ETC both directly through ROS and indirectly through lipid peroxidation products. Moreover, studies in vertebrate models with altered ETC components generally show negative impacts on lifespan and health span, though some exceptions exist [49,50]. Additionally, evidence suggests that declines in heart function with age may be linked to oxidative damage caused by ROS produced during mitochondrial oxidative phosphorylation. One study found that aging impacts the expression of genes involved in ROS production and clearance in both human and rat hearts. In human myocardium, downregulation is observed only for complex I [51]. Given that complex I and complex III are key sources of ROS, this selective downregulation highlights how aging affects mitochondrial function. As primary ROS producers, mitochondria are particularly vulnerable to oxidative damage [52].

In our study, we found elevated levels of OGG1, APE1, and POLG in the older age group, confirming the activation of base excision repair (BER) in response to oxidative damage within mtDNA. Notably, the activity of OGG1 was approximately four times higher than that of APE1, suggesting that APE1 enhances OGG1 activity. With a similar amount of APE1 present, OGG1 activity can increase up to five-fold. Excessive or sustained BER activation has been implicated in age-related pathological processes, including vascular inflammation, endothelial dysfunction, and arterial stiffening, which are subclinical markers of CVD. OGG1 initiates the removal of 8-oxoguanine lesions, while APE1 processes abasic sites and facilitates repair completion. The upregulation of these enzymes reflects a compensatory mechanism in aged individuals to counter increased oxidative damage to mtDNA. The DNA repair enzyme OGG1 relates to unhealthy aging in humans, in particular to inflammaging, which is associated with increased levels of TNF-α. Profiles for OGG1 change during unhealthy aging and are related to TNF-α, BMI, and oxidative DNA/RNA damage [53]. Both in vivo and in vitro results support a "toxic oxidation" model in which OGG1 initiates an escalating oxidation-excision cycle that leads to progressive, age-dependent expansion [54]. Deletion of both alleles of APE1 leads to lethality, and deficiency in cells can promote cell death. Unlike most other BER proteins, APE1 expression is correlated with cellular senescence in human aging [55]. Such stress-induced changes in the mitochondrial genome trigger an adaptive response known as the ISR. This pro-survival mechanism, activated by the mitochondria and other organelles to preserve integrity, is closely associated with the induction of ISR [24]. In the present study, the relative quantitative expression of OMA1, DELE1, and HRI genes showed altered profiles in the older age group. The ISR is a conserved adaptive signaling pathway that coordinates cellular responses to various stressors, including mitochondrial perturbations, as often seen with aging. Specifically, under mitochondrial stress, the inner membrane protease OMA1 is activated and cleaves DELE1, which is then translocated to the cytosol. There, DELE1 activates HRI, which phosphorylates eIF2α, leading to a temporary suppression of global protein synthesis while selectively enhancing the translation of stress-responsive genes, such as ATF4. Chronic ISR activation has been associated with endothelial dysfunction, enhanced pro-inflammatory cytokine production, and increased cardiovascular vulnerability, highlighting its role in age-related pathophysiology.

These data align with earlier research, highlighting a relationship between variations in respiratory chain complex activity and disruptions in mitochondrial gene expression across different populations. Due to its lack of protective histones, limited repair capacity, and proximity to ROS, mtDNA is particularly susceptible to oxidative damage. Such impairment can elevate the risk of chronic illness and result in mtDNA depletion. Our study investigates the methylation status of mtDNA regions, revealing significant alterations in mitochondrial methylation profiles associated with aging and other environmental factors. Cross-sectional analyses indicate higher expression levels of DNMT genes in the older group (Group II), suggesting a potential link between mitochondrial damage and increased methylation. Upregulated expression of DNMTs suggests active epigenetic remodeling within mitochondria during aging. This alteration in mtDNA methylation correlates strongly with elevated levels of proBNP and cardiac troponin I, both clinically recognized surrogate markers for cardiac stress and heart failure. Oxidative stress, particularly from agents like hydrogen peroxide, facilitates the recruitment of DNMT1 to chromatin, leading to changes in nDNA methylation [56]. nDNA methylation, governed primarily by DNMT1 and DNMT3A, is a critical epigenetic mechanism that correlates strongly with chronological aging and is influenced by psychosocial, behavioral, and health-related factors. It plays a pivotal role in regulating inflammatory cytokine expression. For instance, increased DNMT1 levels enhance the release of pro-inflammatory cytokines such as TNF-α and IL-6 in response to lipopolysaccharide exposure, while DNMT3A overexpression elevates methylation at the IL-6 promoter in synovial fibroblasts [57,58]. These methylation changes are associated with elevated cytokine levels in older individuals, reflecting a positive correlation with age, which can lead to transcriptional silencing of genes critical for vascular homeostasis. For example, hypermethylation of promoters in endothelial nitric oxide synthase (eNOS) impairs nitric oxide production, contributing to endothelial dysfunction and vascular stiffness. Similarly, methylation of genes regulating antioxidant pathways can exacerbate oxidative stress - a key driver of vascular inflammation. CpG methylation in the promoters of fibrotic genes such as TGF-β1 and collagen subunits has been associated with cardiac fibrosis, leading to reduced myocardial compliance and heart failure. On the other hand, hypomethylation of inflammatory genes (e.g., TNF-α and IL-6) can drive chronic inflammation, promoting atherosclerosis and plaque instability. Histone modifications (e.g., H3K27ac, H3K4me3, and H3K9me3) further modulate chromatin accessibility. Acetylation marks generally promote gene activation, while methylation marks can either activate or repress transcription depending on context. Altered histone marks in vascular smooth muscle cells and cardiac fibroblasts have been linked to phenotypic switching, vascular remodeling, and increased vascular tone - contributing to hypertension and heart failure. Overall, these epigenetic alterations act in concert to reprogram cardiovascular gene expression networks in response to aging and environmental stress, ultimately promoting disease progression. Differentially methylated CpGs and regions, including AHRR, SCARNA6/SNORD39, SNORA20, and F2RL3, further support this correlation [59]. Additionally, lymphocyte mtDNAcn, relative leukocyte telomere length, and mtDNA methylation are dependent on chronological age [60]. Variations in mitomiR expression profiling revealed differences between age groups, highlighting the role of these short RNAs in regulating mitochondrial gene expression and mtDNA epigenetic modifications [61]. In our study, we observed that upregulation of mitomiR-24 has been implicated in regulating mitochondrial apoptosis pathways and may contribute to reduced mitochondrial turnover and increased cell susceptibility to stress. mitomiR-34a, a well-characterized aging-associated miRNA, negatively regulates SIRT1 and PGC-1α, leading to impaired mitochondrial biogenesis and increased oxidative stress. mitomiR-150 is involved in regulating mitochondrial metabolism and immune signaling, and its elevated levels have been associated with reduced mitochondrial efficiency and chronic inflammation. Finally, mitomiR-155 plays a role in modulating mitochondrial inflammatory signaling and ROS production and is known to promote mitochondrial dysfunction in cardiovascular and metabolic diseases. The collective upregulation of these mitomiRs in aged individuals points toward a coordinated regulatory shift contributing to mitochondrial impairment, altered bioenergetics, and increased cardiovascular vulnerability with age. Further analysis showed that Group II is associated with increased post-translational histone modifications, indicating that errors in epigenetic systems - which control various aspects of chromatin - can impact the transcriptional status of essential genes and contribute to disease. Specifically, we observed elevated levels of H3K4me1 (monomethylation at lysine 4), H3K9ac (acetylation at lysine 9), and H3S10ph (phosphorylation at serine 10) in the older age group. These modifications are commonly linked to active transcriptional states. Furthermore, we detected significant increases in di-methylation at H3K9 and tri-methylation at H3K4 and H3K79, which are known to regulate genes involved in mitochondrial function, inflammation, and cardiovascular homeostasis. These alterations in histone marks suggest that epigenetic remodeling with age may impact the expression of critical genes, thereby contributing to endothelial dysfunction.

Our observations, with respect to the level of proinflammatory cytokines, showed elevated levels in the older individuals, indicating a positive correlation with age. Numerous studies have linked aging to elevated levels of circulating cytokines and proinflammatory markers. This is largely attributed to immunosenescence and increased cytokine secretion by adipose tissue, contributing to chronic inflammation, a phenomenon known as "inflammaging." Elevated levels of interleukin IL-6, IL-1, and TNF-α are associated with higher morbidity and mortality in older adults [62-64], with TNF-α and IL-6 specifically identified as markers of frailty [65-67]. Proinflammatory cytokines also elevate the risk of cardiovascular events through mechanisms such as platelet and endothelial activation [68]. Additionally, mitochondrial ROS are known to activate NRF2, which binds to antioxidant response elements (AREs) in gene promoters to trigger a protective response. This NRF2-mediated response is influenced by redox-sensitive thiol(ate) and NF-κB transcription factors, impacting mitochondrial physiology and structural integrity. Given that older individuals show aberrant inflammatory responses, with increased levels of cytokines like IL-6, TNF-α, and IFN-γ, the activation of NF-κB may further amplify the release of these proinflammatory cytokines, linking mitochondrial ROS, NRF2 activation, and chronic inflammation in aging. 

Beyond the primary findings, this study also revealed a significant correlation between mtDNA methylation and TERT expression, highlighting its potential relevance to CVD risk in the context of aging. Our observations indicate a positive correlation between mtDNA methylation and advanced age, suggesting that increased mtDNA methylation may be associated with both aging and heightened cardiovascular risk. To support this, a previous study has reported that mitochondrial methylation increases with age in humans and in several tissues that have been measured [69]. Further, in a study performed in the peripheral blood of 381 individuals ranging from 38 to 107 years of age, methylation levels of the MT-RNR1 gene were positively associated with increasing age [70,71]. Mitochondrial dysfunction significantly impacts cardiac health through mutations in key mitochondrial genes. Genes such as MT-ND1, MT-ND5, MT-CO1, and tRNAs like MT-TL1 and MT-TH are involved in the ETC and mitochondrial protein synthesis. Their dysfunction impairs ATP production and calcium handling, which are essential for cardiac contractility. Additionally, nuclear genes like OPA1, Drp1, Mfn1, and Mfn2 regulate mitochondrial fusion and fission, and their altered expression disrupts mitochondrial morphology and dynamics, contributing to cardiomyopathy, arrhythmias, and overall cardiac dysfunction. A parallel correlation exists between TERT activity and mtDNA, which may reflect physiological conditions. Growing research has shown that telomeres and mtDNA are regulated in a coordinated manner [72,73]. This joint regulation is crucial for understanding chronic diseases and the aging process [74,75]. Recent reports confirm the interaction between telomeres and mitochondria [76], leading to the proposal of the "telomere-mitochondrial axis" [77] as a target for aging-related molecular damage. Mitochondrial dysfunction contributes to telomere attrition by increasing ROS production, which causes irreversible DNA damage and disrupts normal redox signaling, resulting in oxidative stress. ROS specifically damages telomeric DNA, and telomere attrition is often due to inefficient repair of telomeric DNA single strands. TERT, the catalytic subunit of telomerase, plays a critical role in maintaining telomere length. Downregulation of TERT with age leads to telomere shortening, triggering replicative senescence or apoptosis. This telomere attrition induces oxidative stress, impairing mitochondrial function and increasing ROS production. Mitochondrial dysfunction, in turn, exacerbates oxidative damage to mtDNA, leading to mutations and altered mtDNA methylation patterns. Changes in mtDNA methylation, particularly hypomethylation, can dysregulate mitochondrial gene expression, impair energy production, and activate pro-inflammatory pathways. This cycle of telomere shortening, mitochondrial dysfunction, and epigenetic modifications creates a feedback loop that accelerates aging and contributes to the pathogenesis of CVDs. This connection is supported by research showing that telomere attrition in CD8+ T cells and murine embryos can be mitigated by ROS scavengers and antioxidants, respectively [78,79]. A significant negative correlation was seen in TERT activity with aging, which is supported by the above observations on telomere attrition.

With respect to the potential of mtDNA methylation as a biomarker for CVD, our regression analysis revealed a modest increase in proBNP levels associated with mtDNA methylation. This finding suggests a potential link between mtDNA modifications and cardiovascular conditions. Elevated proBNP levels, a biomarker for heart failure and other cardiovascular disorders, in the context of altered mtDNA methylation, imply that mitochondrial dysfunction may contribute to cardiovascular stress. Previous studies have highlighted changes in the mitochondrial epigenome among individuals with CVD [80]. With advancing age, increased oxidative stress and chronic low-grade inflammation stimulate the upregulation of DNMTs, particularly within the mitochondrial matrix. This leads to hypermethylation at CpG and non-CpG sites within regulatory regions of mtDNA, especially the D-loop and promoters of key respiratory genes (e.g., ND6, COX1, and ATP6/8). Such methylation events disrupt mitochondrial transcription factor binding (e.g., TFAM), impairing transcription and replication of mitochondrial genes essential for ETC integrity and ATP production. The resulting mitochondrial bioenergetic failure elevates ROS generation and triggers compensatory stress signaling pathways, including the mitochondrial unfolded protein response (UPR^mt) and the ISR. This cascade ultimately leads to cardiomyocyte dysfunction and mechanical strain. In response to ventricular wall stress, cardiac myocytes synthesize and secrete BNP, which is cleaved into its biologically active form and the inert prohormone fragment, proBNP. Elevated proBNP levels serve as a functional surrogate for myocardial stress and subclinical heart failure. The observed positive correlation between mtDNA methylation and proBNP in older individuals thus reflects a causal continuum: aging induces mtDNA methylation, which disrupts mitochondrial gene expression, compromises ETC function, increases ROS, leads to cardiomyocyte stress, and results in increased proBNP expression.

Recent research indicates that mtDNA methylation regulates the expression of mitochondrial-derived peptides (MDPs) with cytoprotective functions [81], suggesting that mtDNA methylation levels could reflect overall cellular stress. MDPs are small bioactive peptides encoded within the mitochondrial genome that exert protective effects on cardiovascular function, such as humanin (HN), which acts as a cytoprotective factor by inhibiting apoptosis and oxidative stress in cardiac cells. MOTS-c (Mitochondrial ORF of the 12S rRNA type-c) plays a critical role in regulating metabolic homeostasis, enhancing insulin sensitivity, and reducing inflammation - all of which are key contributors to cardiovascular health. ATP6-derived peptide (ATP6-DP), derived from the mitochondrial ATP synthase subunit 6 gene, is involved in modulating mitochondrial energy output and cellular stress responses. Small humanin-like peptides (SHLPs) maintain mitochondrial integrity, and their loss contributes to fibrosis and endothelial dysfunction; F1-ATPase-derived peptides (F1-DP) ensure energy balance in cardiac cells. Furthermore, mtDNA methylation levels in the blood have been associated with heart rate variability, particularly in individuals exposed to environmental and occupational factors related to CVD [82-84]. These insights further strengthen our hypothesis on the potential of mtDNA methylation as an important biomarker for assessing cardiovascular risk and stress. This study was posted to the medRxiv preprint server on March 15, 2024, titled "Molecular insights into mitoepigenetic stress response signaling in age-associated cardiovascular disease risk" [85].

With the rapid expansion of medical big data, the integration of machine learning in cardiovascular research is gaining momentum. In this study, we observed significant positive correlations between age, mtDNA methylation, and proBNP levels, indicating their potential as predictive biomarkers for CVD. Among the machine learning models tested, the Random Forest classifier demonstrated superior performance, achieving the highest accuracy compared to Logistic Regression, Decision Tree, and SVM. Feature importance analysis consistently identified proBNP and age as the most influential predictors, in agreement with their well-established clinical relevance [86]. The Random Forest model effectively stratified individuals into distinct CVD risk categories based on mtDNA methylation patterns, proBNP concentrations, and age, with elevated values - particularly in older individuals - corresponding to higher risk scores. These findings were reinforced by correlation matrix analyses, highlighting the synergistic role of epigenetic and clinical markers in disease progression. Despite these strengths, the study's limitations, including a modest sample size and a restricted set of input features, underscore the need for future research incorporating larger, gender-specific cohorts and additional variables, such as lifestyle, medical history, and imaging data. Collectively, our results emphasize the importance of a multimodal approach that integrates molecular and clinical biomarkers with machine learning techniques to enhance the early detection and personalized risk prediction of age-related CVD.

## Conclusions

This study highlights the molecular and functional consequences of aging on mitochondrial-mediated epigenetic stress responses and their association with increased CVD risk. We observed significant correlations between age-related mitochondrial alterations - including elevated mtDNA methylation, altered copy number, and telomeric attrition - and markers of mitochondrial dysfunction, underscoring their potential as early biomarkers for CVD risk stratification. The upregulation of mitochondrial stress response genes, coupled with reduced expression of respiratory complex genes in older adults, suggests impaired bioenergetics and enhanced inflammatory signaling with advancing age. Among the machine learning approaches tested, the Random Forest model demonstrated superior predictive performance, supporting its potential utility in identifying individuals at elevated CVD risk based on mitochondrial and metabolic profiles. These findings lay the groundwork for developing targeted interventions, such as mitochondrial antioxidants, senotherapeutics, or epigenetic modulators, to restore mitochondrial health in aging populations. Despite these promising insights, the study’s limitations - including a relatively small sample size and limited population diversity - necessitate further validation in larger, ethnically diverse cohorts. Additionally, translating these findings into clinical practice will require careful consideration of ethical and logistical barriers, including the accessibility and cost of advanced genetic testing. Together, our results provide preliminary, yet compelling, evidence that telomere-mitochondrial interactions and mitochondrial epigenetic remodeling may contribute to age-associated CVD vulnerability, offering a foundation for future precision medicine strategies in cardiovascular aging.
